# Supplementary material for: The moderating and mediating role of eating behaviour traits in acceptance and commitment therapy-based weight management interventions: protocol for an individual participant data meta-analysis
Source: BMJ Open. 2023 Dec 10;13(12):e076411. doi: 10.1136/bmjopen-2023-076411 (PMC10729174; doi:10.1136/bmjopen-2023-076411)
Supplement: Supplementary data [file bmjopen-2023-076411supp001.pdf]

The Moderating and Mediating Role of Eating Behaviour Traits in Acceptance and Commitment Therapy-based Weight Management Interventions: Protocol for an Individual Participant Data Meta-analysis

[SUPPLEMENTARY MATERIAL]

Laura Kudlek [a*], Julia Mueller [a], Patricia Eustacio Colombo [a], Stephen J. Sharp [a] , Simon J Griffin [a, b], Amy Ahern [a]

[a] MRC Epidemiology Unit, School of Clinical Medicine, University of Cambridge, Cambridge, United Kingdom

[b] Primary Care Unit, Department of Public Health and Primary Care, University of Cambridge, Cambridge, United Kingdom

* Corresponding author, email: laura.kudlek@mrc-epid.cam.ac.uk

Contents

1.0 SEARCH STRATEGY 3

1.1 MEDLINE via Ovid 3

1.2 EMBASE via Ovid 3

1.3 AMED via Ovid 3

1.4 CENTRAL via Cochrane 4

1.5 ASSIA via ProQuest 4

1.6 PSYCINFO via ProQuest 5

1.7 WEB OF SCIENCE via web of science 5

1.8 CINAHL via EBSCOhost 5

2.0 DATA EXTRACTION FORM 6

2.1 Top-line details 6

2.2 Study characteristics 6
2.2.1 Methods 6
2.2.2 Notes 6
2.2.3 Participants (at baseline) 7
2.2.4 Interventions 7

2.3 Mediators/Moderators/Outcomes 8

1.0 Search Strategy

We screened studies included in a review by Lawlor et al. (2020) [28] on third wave cognitive behavioural therapies for weight management against this review's eligibility criteria. In addition, we re-ran an adapted search relating to the concepts of (a) ACT and (b) overweight, obesity or weight management from the 25th of September 2019 until the 20th of June 2022. Details of the adapted search strategy are shown below.

1.1 MEDLINE via Ovid

**1**	exp Obesity/ OR exp Overweight/ OR exp Body Weight/ OR exp Body Mass Index/ OR exp Waist Circumference/ OR exp Feeding Behavior/ OR exp Body Weight Changes/ OR exp Caloric Restriction/ OR exp Weight Loss/ OR obes*.mp OR (overweight or over-weight).mp OR (weight adj3 (body or chang* or loss* or maint* or manag* or control* or reduct*)).mp OR (food adj3 (intake or habit*)).mp OR (body mass index or bmi).mp OR body adj3 mass.mp OR (calori* adj3 (restrict* or restrain* or reduc*)).mp OR feeding adj3 behavio*.mp OR (diet* adj3 (restrict* or restrain* or reduc*)).mp OR (waist* adj3 circumferenc*).mp
**2**	"Acceptance and Commitment Therapy"/ OR (acceptance* adj3 (commit* or mind* or base* or focus* or intervention* or therap* or treat*)).mp
**3**	1 AND 2
**4**	Limit 3 to dt=20190925-20220620
**5**	Limit 3 to rd=20190925-20220620
**6**	4 OR 5

1.2 EMBASE via Ovid

**1**	exp Obesity/ OR exp Body Weight/ OR exp Body Mass/ OR exp Waist Circumference/ OR exp Feeding Behavior/ OR exp Caloric Restriction/ OR exp Weight Reduction/ OR obes*.mp OR (overweight or over-weight).mp OR (weight adj3 (body or chang* or loss* or maint* or manag* or control* or reduct*)).mp OR (food adj3 (intake or habit*)).mp OR (body mass index or bmi).mp OR (body adj3 mass).mp OR (calori* adj3 (restrict* or restrain* or reduc*)).mp OR (feeding adj3 behavio*).mp OR (diet* adj3 (restrict* or restrain* or reduc*)).mp OR (waist* adj3 circumferenc*).mp
**2**	"acceptance and commitment therapy"/ OR (acceptance* adj3 (commit* or mind* or base* or focus* or intervention* or therap* or treat*)).mp
**3**	1 AND 2
**4**	Limit 3 to dd=20190925-20220620
**5**	Limit 3 to rd=20190925-20220620
**6**	4 OR 5

1.3 AMED via Ovid

**1**	exp Obesity/ or exp Body Weight/ or exp Body Mass Index/ or exp Weight Loss/ or obes*.mp or (overweight or over-weight).mp or (weight adj3 (body or chang* or loss* or maint* or manag* or control* or reduct*)).mp or (food adj3 (intake or habit*)).mp or (body mass index or bmi).mp or body adj3 mass.mp or (calori* adj3 (restrict* or restrain* or reduc*)).mp or feeding adj3 behavio*.mp or (diet* adj3 (restrict* or restrain* or reduc*)).mp or (waist* adj3 circumferenc*).mp

**2**	(acceptance* adj3 (commit* or mind* or base* or focus* or intervention* or therap* or treat*)).mp
**3**	1 AND 2
**4**	Limit 3 to 2018-Current

1.4 CENTRAL via Cochrane

**#1**	MeSH descriptor: [Obesity] explode all trees	15762
**#2**	MeSH descriptor: [Overweight] explode all trees	18878
**#3**	MeSH descriptor: [Body Weight] explode all trees	31138
**#4**	MeSH descriptor: [Body Mass Index] explode all trees	10927
**#5**	MeSH descriptor: [Waist Circumference] explode all trees	1143
**#6**	MeSH descriptor: [Feeding Behavior] explode all trees	9769
**#7**	MeSH descriptor: [Body Weight Changes] explode all trees	9695
**#8**	MeSH descriptor: [Caloric Restriction] explode all trees	941
**#9**	MeSH descriptor: [Weight Loss] explode all trees	7104
**#10**	obes* in All Text	51603
**#11**	(overweight or over-weight)	19265
**#12**	(weight near/3 (body or chang* or loss* or maint* or manag* or control* or reduct*))	75571
**#13**	(food near/3 (intake or habit*))	11473
**#14**	(body mass index or bmi)	74353
**#15**	(body near/3 mass)	64975
**#16**	(calori* near/3 (restrict* or restrain* or reduc*))	3305
**#17**	feeding near/3 behavio*	5662
**#18**	(diet* near/3 (restrict* or restrain* or reduc*))	15513
**#19**	(waist* near/3 circumferenc*)	10789
**#20**	#1 or #2 or #3 or #4 or #5 or #6 or #7 or #8 or #9 or #10 or #11 or #12 or #13 or #14 or #15 or #16 or #17 or #18 or #19	171531
**#21**	MeSH descriptor: [Acceptance and Commitment Therapy] explode all trees	281
**#22**	(acceptance* near/3 (commit* or mind* or base* or focus* or intervention* or therap* or treat*))	2898
**#23**	#21 or #22	2898
**#24**	#20 and #23	324
	(with Cochrane Library publication date from Aug 2019 to Jun 2022) Apply limits > Select limits > Run search	132

1.5 ASSIA via ProQuest

MAINSUBJECT.EXACT.EXPLODE("Obesity") OR MAINSUBJECT.EXACT.EXPLODE("Body weight") OR MAINSUBJECT.EXACT.EXPLODE("Body Mass Index") OR

MAINSUBJECT.EXACT.EXPLODE("Feeding patterns") OR MAINSUBJECT.EXACT.EXPLODE("Caloric intake") OR obes* OR overweight OR over-weight OR (weight NEAR/3 (body or chang* or loss* or maint* or manag* or control* or reduct*)) OR (food NEAR/3 (intake or habit*)) OR ("body mass index" or bmi) OR (body NEAR/3 mass) OR (calori* NEAR/3 (restrict* or restrain* or reduc*)) OR (feeding NEAR/3 (pattern* or behavio*)) OR (diet* NEAR/3 (restrict* or restrain* or reduc*)) OR (waist* NEAR/3 circumferenc*)
AND
acceptance* NEAR/3 (commit* or mind* or base* or focus* or intervention* or therap* or treat*)
Limit by publication date (2019-09-25 to 2022-06-21)

1.6 PSYCINFO via ProQuest

MAINSUBJECT.EXACT.EXPLODE("Obesity") OR MAINSUBJECT.EXACT.EXPLODE("Body Weight") OR MAINSUBJECT.EXACT.EXPLODE("Body Mass Index") OR MAINSUBJECT.EXACT.EXPLODE("Eating Behavior") OR MAINSUBJECT.EXACT.EXPLODE("Food Intake") OR MAINSUBJECT.EXACT.EXPLODE("Diets") OR obes* OR overweight OR over-weight OR (weight NEAR/3 (body or chang* or loss* or maint* or manag* or control* or reduct*)) OR (food NEAR/3 (intake or habit*)) OR ("body mass index" or bmi) OR (body NEAR/3 mass) OR (calori* NEAR/3 (restrict* or restrain* or reduc*)) OR (feeding NEAR/3 (pattern* or behavio*)) OR (diet* NEAR/3 (restrict* or restrain* or reduc*)) OR (waist* NEAR/3 circumferenc*)
AND
MAINSUBJECT.EXACT.EXPLODE("Acceptance and Commitment Therapy") OR acceptance* NEAR/3 (commit* or mind* or base* or focus* or intervention* or therap* or treat*)
Filtered by 2019-09-25 - 2022-06-21

1.7 WEB OF SCIENCE via web of science

(TS=(obes*) OR TS=(overweight or over-weight) OR TS=(weight NEAR/3 (body or chang* or loss* or maint* or manag* or control* or reduct*)) OR TS=(food NEAR/3 (intake or habit*)) OR TS=("body mass index" or bmi) OR TS=(body NEAR/3 mass) OR TS=(calori* NEAR/3 (restrict* or restrain* or reduc*)) OR TS=(feeding NEAR/3 behavio*) OR TS=(diet* NEAR/3 (restrict* or restrain* or reduc*)) OR TS=(waist* NEAR/3 circumferenc*))
AND
(TS=(acceptance* NEAR/3 (commit* or mind* or base* or focus* or intervention* or therap* or treat*)))
Refined by: PUBLICATION date (2019-09-25 to 2022-06-20)

1.8 CINAHL via EBSCOhost

((MH "Obesity+") OR (MH "Body Weight+") OR (MH "Eating Behavior+") OR (MH "Body Weight Changes+") OR (MH "Weight Loss+") OR (MH "Body Mass Index") OR (MH "Waist Circumference") OR (MH "Weight Reduction Programs") OR (TX obes*) OR (TX (overweight or over-weight)) OR (TX (weight N3 (body or chang* or loss* or maint* or manag* or control* or reduct*))) OR (TX (food N3 (intake or habit*))) OR (TX (body mass index or bmi)) OR (TX (body N3 mass)) OR (TX (calori* N3 (restrict* or restrain* or reduc*))) OR (TX (feeding N3 behavio*)) OR (TX (diet* N3 (restrict* or restrain* or reduc*))) OR (TX (waist* N3 circumferenc*)))
AND
((MH "Acceptance and Commitment Therapy") OR (TX (acceptance* N3 (commit* or mind* or base* or focus* or intervention* or therap* or treat*))))
Limiters - Published Date: 20190801-20220631

2.0 Data extraction form

2.1 Top-line details

ID of data extractor	
Covidence ID	
References (Author, Publication year, Titles)	
Trial ID (if provided)	

2.2 Study characteristics

2.2.1 Methods

Design		
Study start date		
Study end date		
Recruitment i.e. where and how recruited (online, proactive telephone etc)		
Setting (e.g. clinical, commercial, workplace, etc.)		
Country		
Inclusion/exclusion criteria	Inclusion criteria	
	Exclusion criteria	
Inclusion based on specific population characteristic? (e.g. pre-existing condition, gender, ethnicity)		

2.2.2 Notes

Funding statement (copy verbatim)	
Declarations of interest statement (copy verbatim)	
Any other notes to be included in characteristics of included studies table	

2.2.3 Participants (at baseline)

Total N randomized	
N per arm/group (where relevant)	
Total % Female	
Total mean age	
Total mean baseline BMI	
Total mean baseline EE (emotional eating)	
Total mean baseline UE (uncontrolled eating/ disinhibition)	
Total mean baseline R (restraint)	
Total mean baseline other EBTs (rename as appropriate)	

2.2.4 Interventions

(where multiple arms, copy and paste for each arm)

Any shared aspects between intervention and control groups	Target behaviour (e.g. physical activity or nutrition)	
	Mode of Delivery (e.g. group vs individual, online vs in person)	
	Duration & Frequency & Intensity (Dose)	
	Shared Intervention Content/ Components	
Comparison Arm (rename as needed)	Target behaviour (e.g. physical activity or nutrition)	
	Mode of Delivery (e.g. group vs individual, online vs in person)	
	Duration & Frequency & Intensity (Dose)	
	Behaviour change strategies	
	Other intervention details	
Intervention Arm (rename as needed)	Target behaviour (e.g. physical activity or nutrition)	
	Mode of Delivery (e.g. group vs individual, online vs in person)	

	Duration & Frequency & Intensity (Dose)	
	Behaviour change strategies	
	ACT components (add key terms, e.g. cognitive defusion, urge surfing, values, etc.)	
	Other intervention details	

2.3 Mediators/Moderators/Outcomes

How EBTs were measured/defined (which questionnaire was used, with citation)	
When EBTs were measured	
How weight outcome was measured (e.g. self-report vs objective etc.)	
When outcome was measured	
