# Supplementary material for: The moderating and mediating role of eating behaviour traits in acceptance and commitment therapy-based weight management interventions: protocol for an individual participant data meta-analysis
Source: BMJ Open. 2023 Dec 10;13(12):e076411. doi: 10.1136/bmjopen-2023-076411 (PMC10729174; doi:10.1136/bmjopen-2023-076411)
Supplement: Author's manuscript [file bmjopen-2023-076411.draft_revisions.pdf]

BMJ Open is committed to open peer review. As part of this commitment we make the peer review history of every article we publish publicly available.

When an article is published we post the peer reviewers' comments and the authors' responses online. We also post the versions of the paper that were used during peer review. These are the versions that the peer review comments apply to.

The versions of the paper that follow are the versions that were submitted during the peer review process. They are not the versions of record or the final published versions. They should not be cited or distributed as the published version of this manuscript.

BMJ Open is an open access journal and the full, final, typeset and author-corrected version of record of the manuscript is available on our site with no access controls, subscription charges or pay-per-view fees (http://bmjopen.bmj.com).

If you have any questions on BMJ Open's open peer review process please email info.bmjopen@bmj.com

The Moderating and Mediating Role of Eating Behaviour Traits in Acceptance and Commitment Therapy-based Weight Management Interventions: Protocol for an Individual Participant Data Meta-analysis

Journal:	*BMJ Open*
Manuscript ID	bmjopen-2023-076411
Article Type:	Protocol
Date Submitted by the Author:	07-Jun-2023
Complete List of Authors:	Kudlek, Laura ; University of Cambridge, MRC Epidemiology Unit<br>Mueller, Julia; University of Cambridge, MRC Epidemiology unit<br>Eustacio Colombo, Patricia ; University of Cambridge, MRC Epidemiology Unit<br>Sharp, Stephen; University of Cambridge, MRC Epidemiology Unit<br>Griffin, Simon; University of Cambridge<br>Ahern, Amy; University of Cambridge, MRC Epidemiology Unit
Keywords:	Obesity, NUTRITION & DIETETICS, PREVENTIVE MEDICINE, MENTAL HEALTH

SCHOLARONE™
Manuscripts

The Moderating and Mediating Role of Eating Behaviour Traits in Acceptance and Commitment Therapy-based Weight Management Interventions: Protocol for an Individual Participant Data Meta-analysis

Laura Kudlek*, Julia Mueller, Patricia Eustacio Colombo, Stephen J. Sharp, Simon J Griffin, Amy Ahern

Corresponding autor:

Laura Kudlek
laura.kudlek@mrc-epid.cam.ac.uk
MRC Epidemiology Unit
University of Cambridge

For peer review only

For peer review only - http://bmjopen.bmj.com/site/about/guidelines.xhtml

1. Abstract

Introduction: Precision medicine approaches to obesity aim to maximise treatment effectiveness by matching weight management interventions (WMIs) to characteristics of individuals, such as eating behaviour traits (EBTs). Acceptance and Commitment Therapy (ACT) - based WMIs may address EBTs such as emotional and uncontrolled eating more effectively than standard WMIs, and might be most effective in people with high levels of these EBTs. However, few studies have examined this directly. We will examine (1) whether ACT-based WMIs are more effective for people with certain levels of EBTs (i.e. moderation) and (2) whether ACT-based WMIs operate through changes in EBTs (i.e. mediation).

Methods and analysis: This systematic review and Individual Participant Data (IPD) meta-analysis will follow the Preferred Reporting Items for a Systematic Review and Meta-analysis of Individual Participant Data (PRISMA-IPD) guidance. We will include studies on ACT-based WMIs that assessed EBTs in people with a body mass index ≥25kg/m^2. We identified studies by screening studies included in a previous review (Lawlor et al., 2020) of third wave cognitive behavioural WMIs, and updating the search to 20.06.2022. We will request IPD from eligible published and unpublished studies. We will harmonise and re-analyse IPD using a two-stage random effects meta-analysis pooling within-trial interactions to investigate moderating effects and using a one-stage simultaneous equation model to examine mediating effects. We will assess the risk of bias of included studies using the Cochrane Risk of Bias tool 2 (RoB2) and the Risk of Bias in Non-randomized Studies of Interventions tool (ROBINS-I).

Ethics and Dissemination: Collation and secondary analysis of pseudonymized IPD does not require renewed ethical approval. IPD sharing will follow data transfer agreements and co-authorship will be offered to investigators contributing IPD. Findings will be disseminated through peer-reviewed journals and conferences and will contribute to the lead author's PhD thesis.

PROSPERO Registration Number: CRD42022359691

For peer review only

2. Strengths and Limitations

- To the best of our knowledge, this will be the first individual participant data meta-analysis exploring the role of eating behaviour traits in acceptance and commitment-based weight management interventions.
- Obtaining, harmonising and re-analysing individual participant data from systematically identified studies allows us to investigate questions around mediation and moderation that individual studies are typically not powered to detect.
- Individual participant data might not be received from all eligible trials due to unresponsiveness, inability or unwillingness to share data and complex data sharing procedures. This might introduce a possibility for bias.
- Individual studies are likely to exhibit heterogeneity in study populations, nature and intensity of interventions, duration of follow-up, outcome assessment and measures of EBTs, which may challenge the interpretation of data from pooled analyses and necessitate sensitivity analyses.

For peer review only

For peer review only - http://bmjopen.bmj.com/site/about/guidelines.xhtml

3. Introduction

Obesity is associated with health risks, such as diabetes, metabolic diseases, and several types of cancer [1]. Worldwide prevalence of overweight and obesity has increased almost threefold since 1975 [1] and is now estimated to be around 60% in the United Kingdom [2] and 70% in the United States [3]. Behavioural weight loss treatments can achieve weight loss, leading to meaningful improvements in health, such as a reduced likelihood of developing diabetes [4–6]. However, outcomes of obesity treatments vary highly among individuals, with some individuals losing significantly more weight than others, and only a proportion of people maintaining weight loss [7]. Precision medicine aims to understand what factors contribute to individual differences in treatment response, and how future intervention content and delivery can be adapted accordingly to maximise their net benefit [8,9]. Eating behaviour traits (EBTs) may be such a factor [10–14]. EBTs are defined as personal tendencies regarding an individual's reaction to food and food-related cues that determine the type, amount, and frequency of food consumed, as well as when to start and stop eating [15,16]. Commonly measured EBTs are restraint (the conscious control of food intake to influence body weight), uncontrolled eating (the tendency to overeat in response to feelings of hunger and external stimuli) and emotional eating (the tendency to overeat in response to negative emotions) [17–19].

During behavioural weight management interventions (WMIs), restraint typically increases and uncontrolled and emotional eating decrease [20–23]. Standard behavioural WMIs involve dietary and physical activity advice, as well as behaviour change techniques such as self-monitoring, goal setting, planning, problem solving, and cognitive restructuring [24]. However, individuals higher in emotional and uncontrolled eating might benefit from additional support targeting these EBTs [10,25,26]. Acceptance and commitment therapy (ACT)-based WMIs train the recognition of food cues or triggers of overeating as well as the acceptance of unpleasant sensations (e.g., cravings or uncomfortable emotions). This can reduce the tendency to rely on food to relieve urges and regulate emotions, and is thus theorised to be more relevant for emotional and uncontrolled eating than standard cognitive behavioural techniques. A recent systematic review and meta-analysis concluded that ACT-based WMIs are effective at reducing emotional eating [27], and a systematic review and network meta-analysis found ACT-based WMIs to be most effective at changing weight outcomes compared to other third wave cognitive behavioural therapies [28].

However, to determine how useful EBTs and ACT-based WMIs could be for precision medicine approaches, it is also important to understand (a) whether ACT-based WMIs are more effective for people high in emotional and/or uncontrolled eating (i.e., EBTs as effect moderators) and (b) whether changes in EBTs are mechanisms of effectiveness by which ACT-based WMIs impact weight outcomes (i.e., EBTs as effect mediators). Although decreases in emotional and uncontrolled eating have been associated with improved weight loss in a variety of interventions [12,29–32], few studies of ACT-based WMIs have examined the role of EBTs in detail. While studies of ACT-based WMIs often measure EBTs, they are mostly reported as outcomes, finding changes in EBTs in the desired direction [33]. Forman et al. additionally explored the moderating role of EBTs, finding ACT-based

For peer review only

interventions to be more effective than standard behavioural treatment in people with high initial levels of emotional eating in one study [34], but not in another [35]. However, sample sizes of these studies were small (N=128 and N=190), and thus likely underpowered for the detection of moderating effects. To our knowledge, no studies of ACT-based interventions have yet examined mediating effects of changes in EBTs.

The scarcity of existing research that has directly examined the mediating and moderating role of EBTs prevents the synthesis of findings in a traditional meta-analysis and makes it difficult to form comprehensive conclusions. Individual Participant Data (IPD) meta-analysis allows us to collate and reanalyse raw data from all relevant studies that have measured the variables of interest, regardless of whether or not the studies have analysed or reported relationships of interest in the original publications [36]. This is likely to increase the number of included studies when compared to a traditional aggregate meta-analysis. Additionally, heterogeneity is reduced by harmonising data from different measures and re-analysing IPD from original studies according to a uniform analysis plan [36]. Using an IPD meta-analysis thus increases the quantity and quality of data on EBTs in ACT-based WMIs beyond existing evidence, generating a resource to address questions around the moderating and mediating role of EBTs in ACT-based WMIs. This will provide us with new understanding about the potential to use ACT-based interventions to support people with high levels of EBTs as part of a precision medicine approach to obesity treatment that directs specific interventions to those that are most likely to benefit from them.

3.1 Objectives

The aim of this IPD meta-analysis is to obtain and re-analyse IPD from studies assessing EBTs in ACT-based WMIs to examine:

(a) to what extent the effect of ACT-based WMIs on weight loss depends on individuals' levels of baseline emotional and uncontrolled eating
(b) to what extent changes in EBTs (restraint, uncontrolled eating, emotional eating) mediate the effect of ACT-based WMIs on weight loss

4. Methods and analysis

This project will follow guidance from the Preferred Reporting Items for Systematic Reviews and Meta-Analyses for IPD extension (PRISMA-IPD).[37] The protocol will additionally follow the PRISMA protocol extension (PRISMA-P) where applicable. We will communicate the current stage of work on the project by using either past or future tense to describe relevant sections.

For peer review only - http://bmjopen.bmj.com/site/about/guidelines.xhtml

4.1 Study identification

4.1.1 Eligibility criteria

Studies were considered eligible for inclusion if they met the following criteria:

- **Population:** Community-dwelling adults (aged 18 and older) with a BMI ≥ 25 kg/m^2. Studies were excluded if participants were recruited purely based on having a chronic disease or being pregnant.
- **Intervention:** Interventions with the primary goal of weight loss or weight loss maintenance (referred to as weight management interventions in the remainder of this protocol) that report incorporating strategies based on ACT. ACT-based interventions from different contexts were eligible (e.g. online, in person, health care setting, commercial), and they were eligible either as standalone treatment or as part of a wider weight management intervention.
- **Comparators:** No/ wait-list control, minimal intervention (e.g. leaflet, brief advice), or standard behavioural WMI. Studies with no control group will be eligible for a subset of analyses.
- **Outcome:** Weight assessed at post-treatment or post-treatment and any follow-up point. A follow-up point of at least 3-months post-baseline had to be available.
- **Mediators/ Moderators:** Eating behaviour traits assessed at baseline, post-treatment or both. Disordered eating will be excluded.
- **Study design:** Due to the scarcity of research in the field, we will include all types of intervention studies to obtain as much IPD as possible (randomised controlled trials (RCTs), non-RCTs, prospective cohort and case series studies). However, only RCTs and cluster-RCTs will be eligible for the full set of analyses.

4.1.2 Search strategy

We screened studies included in a review by Lawlor et al. (2020) [28] on third wave cognitive behavioural therapies for weight management against this review's eligibility criteria. In addition, we re-ran an adapted search relating to the concepts of (a) ACT and (b) overweight, obesity or weight management from the 25th of September 2019 until the 20th of June 2022 (see Appendix 1). Concepts relating to other third wave cognitive behavioural therapies were deleted from the original search strategy to match the narrower focus of this IPD meta-analysis. No restrictions on language were applied. We searched the databases MEDLINE, CINAHL, Embase, PsycINFO, AMED, ASSIA, Web of Science, the Cochrane database (CENTRAL) and hand-searched the reference lists of key publications. Appendix 1 depicts the search strategy as applied to MEDLINE. Furthermore, when requesting IPD, all authors of included studies will be asked whether they are aware of any additional eligible published or unpublished data.

For peer review only - http://bmjopen.bmj.com/site/about/guidelines.xhtml

4.1.3 Study selection

Two independent reviewers, PEC and LK, screened both title and abstracts and full texts in Covidence [38]. Disagreements were resolved by discussion and consulting of a third reviewer, AA, where necessary. We will contact authors of protocols and conference abstracts to resolve any outstanding questions on eligibility and to enquire about their willingness to share unpublished results.

4.2 Risk of bias assessment

The Cochrane Risk of Bias tool 2 (RoB2)[39] or the Risk of Bias in Non-randomised Studies of Interventions tool (ROBINS-I)[40] will be used by two researchers independently to assess risk of bias. Disagreements will be resolved by discussion and consulting of a third reviewer if necessary.

4.3 Variables requested

The following variables will be requested in a detailed data dictionary, including definitions.

- De-identified participant ID
- Age at Baseline
- Sex
- Height at baseline
- Weight at baseline, end of intervention and any follow-up
- Allocated trial arm
- Number of sessions attended
- All measured EBTs at baseline, end of intervention and any follow up point
- All measures of experiential avoidance and/or psychological flexibility at baseline, end of intervention, and any follow-up point
- Variables that describe which participants were excluded from main analysis and why

4.4 Data collection and management

4.4.1 Extraction of published data on study characteristics

Data on study characteristics will be extracted from published manuscripts by two reviewers independently, using a form that will be adapted from the Cochrane data extraction form [41]. These include data on study design and setting, participant sociodemographic characteristics (e.g. ethnicity, education, socioeconomic status) and details on the intervention and control conditions. A third

For peer review only

reviewer will cross-check extractions for any discrepancies, and original study authors will be provided with an opportunity to cross-check extractions for accuracy, including any potential updates.

4.4.2 Requesting IPD

Authors will be contacted via email, outlining the purpose of the study, providing the study protocol, and asking them to collaborate on the project by providing IPD. If the corresponding authors agree, more detailed instructions including an outline of the specific data requested and a data dictionary will be shared. If authors are not able to share their data, we will ask them to perform the analyses using a pre-specified protocol and share the results so that we can include them in the meta-analysis. Authors will be sent two reminders after initial contact, each with a time-period of around three weeks between them. If no response is given and published results are not suitable for meta-analysis, the study concerned will be excluded. Studies that are excluded due to inability to contact will be summarised in a table in the supplementary materials of the publication.

4.4.3 Collecting IPD

Data will be accepted in any format, but a Microsoft Excel format is preferred. A data dictionary will be provided prior to data transfer. Any data sharing will strictly follow the conditions pre-specified in data transfer agreements, and all data will be shared via, and stored in, the MRC Epidemiology Unit's secure research drive. After receiving data, a copy will be saved that will be kept as original. Working files will be converted and imported into R version 4.1.2 [42].

4.4.4 Data harmonisation

Data will be harmonised according to the pre-specified data dictionary to merge it into a combined dataset. Variables will be recoded and transformed using a uniform coding scheme (e.g. height and weight will be transformed into metric units, age will be converted into age in years, etc.). EBTs are likely to have been assessed using different questionnaires across studies (e.g. TFEQ-R18, TFEQ-51, DEBQ, etc.). To harmonise EBT data, we will standardise EBT values. To facilitate this, we will obtain item-level data as well as subscale scores.

4.4.5 Data checking

Data received will be compared to that reported in the original publication by running descriptive statistics (e.g. sample size, weight loss outcomes). Additionally, if both item and subscale-levels of EBTs are provided, subscales will be re-computed and compared to those provided to confirm item scoring and ensure the uniform allocation of items to subscales. In case of any discrepancies, authors

For peer review only - http://bmjopen.bmj.com/site/about/guidelines.xhtml

will be contacted for clarification. If discrepancies cannot be resolved, the nature and severity of the deviation will be evaluated to determine whether the study can be included. After a study has been checked, a summary will be sent to the original authors to give them the opportunity to address any issues before merging the study into the pooled dataset.

4.4.6 Database creation and aggregation

A merged dataset containing harmonised data from all included studies will be created for convenience when managing and analysing data. Data from different studies will be distinguished using a unique identifier for each study that is allocated before the final merging. To check for any errors introduced during the merging phase, descriptive statistics of each study will be run before merging datasets and compared to corresponding statistics produced after merging.

4.4.7 Studies where IPD is not available

If authors are unable to share their raw data, we will ask them to perform the required analyses for us and share their results (i.e. a federated approach). This will allow us to still include the study in the moderation analyses. To check whether this might introduce bias, we will conduct sensitivity analysis using only the data from studies providing IPD. If authors can neither provide IPD, nor provide the outcome statistics of requested analyses (i.e. federated data), any published data will be synthesised and compared to included studies. This will include available published data on sociodemographic variables, such as socioeconomic status, sex, and age, as well as any reported data on eating behaviours at baseline and end of intervention, and weight data at baseline, end of intervention and follow-up. As with data on study characteristics, published data on EBTs and weight outcomes will be extracted in duplicate using a pre-specified data extraction form based on the Cochrane data extraction form template [41].

4.5 Data analyses

4.5.1 Descriptive statistics

Descriptive statistics and the proportion of missing values of age, sex, baseline EBTs and baseline BMI will be derived from the raw data directly. These will be reported for each study, as well as for the overall sample included in the meta-analyses. Other study characteristics, of which IPD will not be requested, e.g. SES and ethnicity, will be extracted from published reports.

4.5.2 Statistical analysis

For peer review only

To gain a more complete picture of the role of EBTs in WMIs, we will explore to what extent (1) baseline EBTs are associated with changes in weight over the intervention and follow-up period, and (2) to what extent changes in EBTs are associated with changes in weight over the intervention and follow-up period before conducting the main moderation and mediation analyses. These initial associations will be explored using linear regression, adjusted for study arm, age, sex, baseline weight and duration of follow up. This will allow us to incorporate evidence from studies without a control group. Findings will be synthesised using a two-stage IPD meta-analysis approach [36], in which analyses are first performed for each study individually, and then the results are combined using random effects meta-analysis. This allows for the incorporation of studies that do not provide IPD if collaborators perform requested analyses at their home institutions and share only their output.

4.5.2.1 Moderation

To investigate whether the effect of ACT-based interventions on weight depends on people's levels of emotional and uncontrolled eating, we will use a two-stage IPD-MA approach and perform a random-effects meta-analysis to combine within-trial regression coefficients of the intervention*EBT interactions. To facilitate interpretation of potential interaction effects with EBT as a continuous variable, we will also fit regression models in three EBT subgroups (reflecting "low", "medium", and "high" levels of the respective EBT) in each trial and combine the estimated subgroup-specific effects using random-effects meta-analysis. Subgroups will be determined by using the tertile EBT scores of the pooled sample as cut-offs for each individual trial. We will investigate the potential moderating effects of EBTs on weight loss outcomes at the end of the intervention as well as at available follow-up timepoints (e.g. 6 and 12 months).

4.5.2.2 Mediation

Mediation analyses will follow 'A Guideline for Reporting Mediation Analyses' (AGReMA) guidelines [43]. We will use the simultaneous equations model (SEM) approach outlined by Huh et al. 2022 [44] to estimate (a) the direct effect by which ACT-based interventions impact weight loss, (b) the indirect effect by which they impact weight loss via changes in EBTs, (c) and the total effect by which weight loss is impacted. We will estimate direct, indirect and total effects on weight loss outcomes at the end of the intervention as well as at available follow-up timepoints (e.g. 6 and 12 months). Figures 1-2 depict the path diagrams that will be tested both in each individual trial, i.e. "study-specific sub-models", and in a pooled sample using a one-stage approach, i.e. "overall model". The SEM for the overall models will control for clustering within studies by using complex survey analysis. While the one-stage approach eliminates the risk of aggregation/ ecological bias, it requires IPD, making a federated approach (where authors share only their output) impossible. Thus, studies that cannot provide IPD will be excluded for this part of the analysis. If trials with less than 50 participants per

For peer review only - http://bmjopen.bmj.com/site/about/guidelines.xhtml

For peer review only

intervention arm will be included, we will consider adapting the path diagram depicted in Figures 1 and 2 by adding the variables age and height with a path to both the outcome and intervention arm to control for possible imbalances between the randomised groups. If it is concluded from previous moderation analyses (Section 4.5.2.1) that ACT-based interventions only have a significant effect on weight outcomes in one or two of the three EBT level subgroups (low, medium or high EBT levels), then we will repeat the mediation analyses in the subgroup(s) that did show a significant effect on weight outcomes. Analyses will be performed in R [42], using the lavaan [45] and lavaan.survey [46] packages.

2.5.3 Sensitivity Analyses

We will conduct a series of sensitivity analyses. At the study-level, we will compare results from analyses performed in (a) the full set of studies versus excluding studies classified at high risk of bias using the RoB2 (also see section *4.2 Risk of Bias Assessment*) (b) studies providing IPD data versus studies providing federated data only (also see section *4.4.7 Studies where IPD is not available*) (c) studies with waitlist or minimal control conditions versus studies with standard behavioural control conditions (d) studies that significantly reduced experiential avoidance (as this is the hypothesised mechanism of action of ACT-based WMIs) versus studies that did not significantly reduce experiential avoidance. At the individual level, we will compare results from analyses performed in (e) all individuals versus those that received a sufficient dose of the intervention (as determined from liaising with study authors). Details will be described in a separate analysis plan.

4.6 Patient and Public Involvement and Engagement (PPIE)

We will seek PPIE input for the interpretation of analyses and implication of results in the form of remote focus group meetings with two or more PPIE members. This may result in co-authorship on the publication.

5. Ethics and Dissemination

The collation and secondary analysis of pseudonymized IPD does not require new ethical approval. Data from individual trials will be shared with the lead institution under appropriate data sharing agreements. We will disseminate findings through peer-reviewed journals and conferences. Investigators contributing data to this analysis will be offered co-authorship. This study will contribute to the lead author's PhD thesis.

For peer review only

For peer review only - http://bmjopen.bmj.com/site/about/guidelines.xhtml

6. References

1. World Health Organization. Obesity and overweight.

2. NHS digital. Health survey for England 2019. 2020;(December):1-22. www.statisticsauthority.gov.uk/assessment/code-of-practice

3. National Institute of Diabetes and Digestive and Kidney Disease. Overweight & Obesity Statistics. Published 2021. https://www.niddk.nih.gov/health-information/health-statistics/overweight-obesity

4. Twells LK, Harris Walsh K, Blackmore A, et al. Nonsurgical weight loss interventions: A systematic review of systematic reviews and meta-analyses. *Obesity Reviews*. 2021;22(11). doi:10.1111/obr.13320

5. Hartmann-Boyce J, Johns DJ, Jebb SA, Summerbell C, Aveyard P. Behavioural weight management programmes for adults assessed by trials conducted in everyday contexts: Systematic review and meta-analysis. *Obesity Reviews*. 2014;15(11):920-932. doi:10.1111/obr.12220

6. LeBlanc ES, Patnode CD, Webber EM, Redmond N, Rushkin M, O'Connor EA. Behavioral and pharmacotherapy weight loss interventions to prevent obesity-related morbidity and mortality in adults updated evidence report and systematic review for the US preventive services task force. *JAMA - Journal of the American Medical Association*. 2018;320(11):1172-1191. doi:10.1001/jama.2018.7777

7. MacLean PS, Wing RR, Davidson T, et al. NIH working group report: Innovative research to improve maintenance of weight loss. *Obesity*. 2015;23(1):7-15. doi:10.1002/oby.20967

8. U. S. Food and Drug Administration (FDA). Precision Medicine. Published 2018. Accessed July 5, 2022. https://www.fda.gov/medical-devices/in-vitro-diagnostics/precision-medicine

9. Bomberg EM, Ryder JR, Brundage RC, et al. Precision medicine in adult and pediatric obesity: a clinical perspective. *Ther Adv Endocrinol Metab*. 2019;10. doi:10.1177/2042018819863022

10. van Strien T. Causes of Emotional Eating and Matched Treatment of Obesity. *Curr Diab Rep*. 2018;18(6). doi:10.1007/s11892-018-1000-x

11. Boutelle KN, Manzano MA, Eichen DM. Appetitive traits as targets for weight loss: The role of food cue responsiveness and satiety responsiveness. *Physiol Behav*. 2020;224(June):113018. doi:10.1016/j.physbeh.2020.113018

12. Braden A, Flatt SW, Boutelle KN, Strong D, Sherwood NE, Rock CL. Emotional eating is associated with weight loss success among adults enrolled in a weight loss program. *J Behav Med*. 2016;39(4):727-732. doi:10.1007/s10865-016-9728-8

13. Boutelle KN, Eichen DM, Peterson CB, et al. Effect of a Novel Intervention Targeting Appetitive Traits on Body Mass Index Among Adults With Overweight or Obesity. *JAMA Netw Open*. 2022;5(5):e2212354. doi:10.1001/jamanetworkopen.2022.12354

For peer review only

14. Niemeier HM, Leahey T, Palm Reed K, Brown RA, Wing RR. An Acceptance-Based Behavioral Intervention for Weight Loss: A Pilot Study. *Behav Ther*. 2012;43(2):427-435. doi:10.1016/j.beth.2011.10.005

15. Llewellyn, Fildes A. Behavioural Susceptibility Theory: Professor Jane Wardle and the Role of Appetite in Genetic Risk of Obesity. *Curr Obes Rep*. 2017;6(1):38-45. doi:10.1007/s13679-017-0247-x

16. Llewellyn C, Wardle J. Behavioral susceptibility to obesity: Gene–environment interplay in the development of weight. *Physiol Behav*. 2015;152:494-501. doi:https://doi.org/10.1016/j.physbeh.2015.07.006

17. Stunkard AJ, Messick S. Eating Inventory. *APA PsychTests*. Published online 1988.

18. Karlsson J, Persson LO, Sjo L, Èm È, Sullivan M. *Psychometric Properties and Factor Structure of the Three-Factor Eating Questionnaire (TFEQ) in Obese Men and Women. Results from the Swedish Obese Subjects (SOS) Study*. Vol 24.; 2000. www.nature.com/ijo

19. Tholin S, Rasmussen F, Tynelius P, Karlsson J. Genetic and environmental influences on eating behavior: the Swedish Young Male Twins Study. *American Journal of Clinical Nutrition*. Published online 2005.

20. Provencher V, Bégin C, Tremblay A, et al. Health-At-Every-Size and Eating Behaviors: 1-Year Follow-Up Results of a Size Acceptance Intervention. *J Am Diet Assoc*. 2009;109(11):1854-1861. doi:10.1016/j.jada.2009.08.017

21. Nurkkala M, Kaikkonen K, Vanhala ML, Karhunen L, Keränen AM, Korpelainen R. Lifestyle intervention has a beneficial effect on eating behavior and long-term weight loss in obese adults. *Eat Behav*. 2015;18:179-185. doi:10.1016/j.eatbeh.2015.05.009

22. Chew HSJ, Lau ST, Lau Y. Weight-loss interventions for improving emotional eating among adults with high body mass index: A systematic review with meta-analysis and meta-regression. *European Eating Disorders Review*. Published online April 23, 2022. doi:10.1002/erv.2906

23. Mason C, de Dieu Tapsoba J, Duggan C, Wang CY, Alfano CM, McTiernan A. Eating behaviors and weight loss outcomes in a 12-month randomized trial of diet and/or exercise intervention in postmenopausal women. *International Journal of Behavioral Nutrition and Physical Activity*. 2019;16(1). doi:10.1186/s12966-019-0887-1

24. National Institute for Health and Care Excellence. Obesity: identification, assessment and management. https://www.nice.org.uk/guidance/cg189/chapter/1-Recommendations#behavioural-interventions.

25. Lillis J, Niemeier HM, Ross KM, et al. Weight loss intervention for individuals with high internal disinhibition: Design of the Acceptance Based Behavioral Intervention (ABBI) randomized controlled trial. *BMC Psychol*. 2015;3(1):1-10. doi:10.1186/S40359-015-0075-2

26. Braden A, Ph D, Flatt SW, et al. Emotional Eating is Associated with Weight Loss Success among Adults Enrolled in a Weight Loss Program. *J Behav Med*. 2016;39(4):727-732. doi:10.1007/s10865-016-9728-8.Emotional

For peer review only

27. di Sante J, Akeson B, Gossack A, Knäuper B. Efficacy of ACT-based treatments for dysregulated eating behaviours: A systematic review and meta-analysis. *Appetite*. 2022;171. doi:10.1016/j.appet.2022.105929

28. Lawlor ER, Islam N, Bates S, et al. Third-wave cognitive behaviour therapies for weight management: A systematic review and network meta-analysis. *Obesity Reviews*. 2020;21(7):1-21. doi:10.1111/obr.13013

29. Keränen AM, Savolainen MJ, Reponen AH, et al. The effect of eating behavior on weight loss and maintenance during a lifestyle intervention. *Prev Med (Baltim)*. 2009;49(1):32-38. doi:10.1016/j.ypmed.2009.04.011

30. Butryn ML, Thomas JG, Lowe MR. Reductions in internal disinhibition during weight loss predict better weight loss maintenance. *Obesity (Silver Spring)*. 2009;17:1101-1103. doi:10.1038/oby.2008.646

31. Nurkkala M, Kaikkonen K, Vanhala ML, Karhunen L, Keränen AM, Korpelainen R. Lifestyle intervention has a beneficial effect on eating behavior and long-term weight loss in obese adults. *Eat Behav*. 2015;18:179-185. doi:10.1016/j.eatbeh.2015.05.009

32. Urbanek JK, Metzgar CJ, Hsiao PY, Piehowski KE, Nickols-Richardson SM. Increase in cognitive eating restraint predicts weight loss and change in other anthropometric measurements in overweight/obese premenopausal women. *Appetite*. 2015;87:244-250. doi:10.1016/j.appet.2014.12.230

33. Mueller J, Richards R, Jones RA, et al. Supporting Weight Management during COVID-19 (SWiM-C): A randomised controlled trial of a web-based, ACT-based, guided self-help intervention. *Obes Facts*. Published online April 13, 2022. doi:10.1159/000524031

34. Forman EM, Butryn ML, Juarascio AS, et al. The mind your health project: A randomized controlled trial of an innovative behavioral treatment for obesity. *Obesity*. 2013;21(6):1119-1126. doi:10.1002/oby.20169

35. Forman EM, Butryn ML, Manasse SM, et al. Acceptance-based versus standard behavioral treatment for obesity: Results from the mind your health randomized controlled trial. *Obesity*. 2016;24(10):2050-2056. doi:10.1002/oby.21601

36. Riley RD, Stewart LA, Tierney JF. *Individual Participant Data Meta-Analysis for Healthcare Research*. Wiley; 2021. doi:10.1002/9781119333784.ch1

37. Stewart LA, Clarke M, Rovers M, et al. Preferred reporting items for a systematic review and meta-analysis of individual participant data: The PRISMA-IPD statement. *JAMA - Journal of the American Medical Association*. 2015;313(16):1657-1665. doi:10.1001/jama.2015.3656

38. Veritas Health Innovation. Covidence systematic review software. Published online 2022. Accessed July 6, 2022. www.covidence.org

39. Higgins J, Savović J, Page M, Elbers R, Sterne J. Chapter 8: Assessing risk of bias in a randomized trial. In: Higgins J, Thomas J, Chandler J, et al., eds. *Cochrane Handbook for Systematic Reviews of Interventions*. version 6.3. Cochrane; 2022. Accessed June 23, 2022. www.training.cochrane.org/handbook

For peer review only

For peer review only - http://bmjopen.bmj.com/site/about/guidelines.xhtml

40. Sterne JA, Hernán MA, Reeves BC, et al. ROBINS-I: a tool for assessing risk of bias in non-randomised studies of interventions. *BMJ*. Published online October 12, 2016:i4919. doi:10.1136/bmj.i4919

41. Cochrane Developmental Psychosocial and Learning Problems. Data Extraction Forms. Published 2014. Accessed June 23, 2022. https://dplp.cochrane.org/data-extraction-forms

42. R Core Team. R: A language and environment for statistical computing. Published online 2021. Accessed June 23, 2022. https://www.R-project.org/

43. Lee H, Cashin AG, Lamb SE, et al. A Guideline for Reporting Mediation Analyses of Randomized Trials and Observational Studies: The AGReMA Statement. *JAMA - Journal of the American Medical Association*. 2021;326(11):1045-1056. doi:10.1001/jama.2021.14075

44. Huh D, Li X, Zhou Z, et al. A Structural Equation Modeling Approach to Meta-analytic Mediation Analysis Using Individual Participant Data: Testing Protective Behavioral Strategies as a Mediator of Brief Motivational Intervention Effects on Alcohol-Related Problems. *Prevention Science*. 2022;23(3):390-402. doi:10.1007/s11121-021-01318-4

45. Rosseel Y. *Journal of Statistical Software Lavaan: An R Package for Structural Equation Modeling*.; 2012. http://www.jstatsoft.org/

46. Oberski D. *Lavaan.Survey: An R Package for Complex Survey Analysis of Structural Equation Models*. Vol 57.; 2014. http://www.jstatsoft.org/

7. Authors Contributions

LK drafted the protocol manuscript, with input from AA, SJS and JM. All authors provided comments on the manuscript at several stages and have read and approved the final version of the manuscript.

8. Funding Statement

This work was supported by the Medical Research Council MC_UU_00006/6 as part of LK's PhD

9. Competing Interest Statement

AA, JM, SJS and SJG were authors of two studies identified as eligible for this IPD meta-analysis (SWiM-F and SWiM-C). JM is a Trustee for the Association for the Study of Obesity (unpaid role). SG has received honoraria from Astra Zeneca for contributing to postgraduate education sessions for primary care teams concerning the management of type 2 diabetes. SG is a trustee of the Novo Nordisk UK Research Foundation. AA is a member of the WW Scientific Advisory Board.

For peer review only

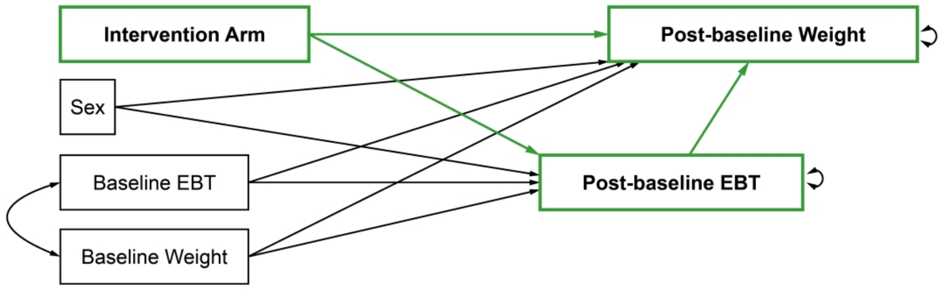

Figure 1: Path diagram for individual EBTs

397x124mm (300 x 300 DPI)

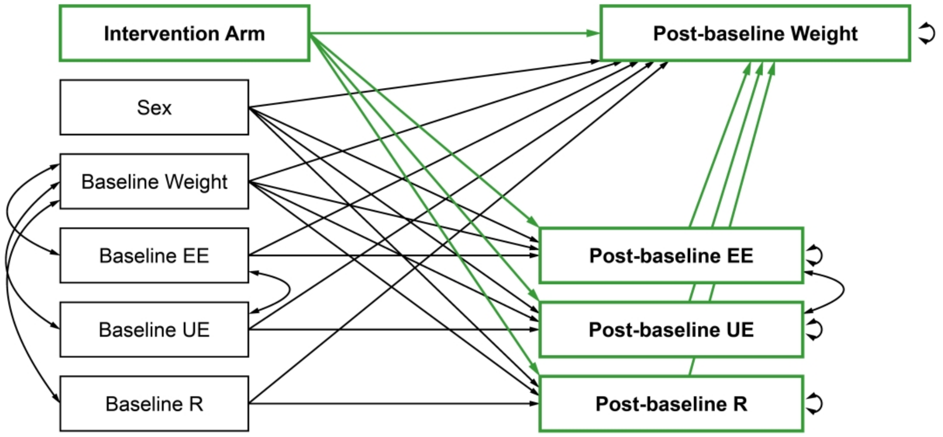

Figure 2: Joint path diagram combining individual EBTs (e.g. emotional eating (EE), uncontrolled eating (UE) and restraint (R))

397x188mm (300 x 300 DPI)

Appendix

1. Adapted search strategy as applied to MEDLINE

**1**	exp Obesity/ OR exp Overweight/ OR exp Body Weight/ OR exp Body Mass Index/ OR exp Waist Circumference/ OR exp Feeding Behavior/ OR exp Body Weight Changes/ OR exp Caloric Restriction/ OR exp Weight Loss/ OR obes*.mp OR (overweight or over-weight).mp OR (weight adj3 (body or chang* or loss* or maint* or manag* or control* or reduct*)).mp OR (food adj3 (intake or habit*)).mp OR (body mass index or bmi).mp OR body adj3 mass.mp OR (calori* adj3 (restrict* or restrain* or reduc*)).mp OR feeding adj3 behavio*.mp OR (diet* adj3 (restrict* or restrain* or reduc*)).mp OR (waist* adj3 circumferenc*).mp
**2**	"Acceptance and Commitment Therapy"/ OR (acceptance* adj3 (commit* or mind* or base* or focus* or intervention* or therap* or treat*)).mp
**3**	1 AND 2
**4**	Limit 3 to dt=20190925-20220620
**5**	Limit 3 to rd=20190925-20220620
**6**	4 OR 5

For peer review only

PRISMA-IPD Checklist of items to include when reporting a systematic review and meta-analysis of individual participant data (IPD)

PRISMA-IPD Section/topic	Item No	Checklist item	Reported on page
**Title**			
Title	1	Identify the report as a systematic review and meta-analysis of individual participant data.	1
**Abstract**			
Structured summary	2	Provide a structured summary including as applicable:	2
		**Background**: state research question and main objectives, with information on participants, interventions, comparators and outcomes.	
		**Methods**: report eligibility criteria; data sources including dates of last bibliographic search or elicitation, noting that IPD were sought; methods of assessing risk of bias.	
		**Results**: provide number and type of studies and participants identified and number (%) obtained; summary effect estimates for main outcomes (benefits and harms) with confidence intervals and measures of statistical heterogeneity. Describe the direction and size of summary effects in terms meaningful to those who would put findings into practice.	
		**Discussion:** state main strengths and limitations of the evidence, general interpretation of the results and any important implications.	
		**Other:** report primary funding source, registration number and registry name for the systematic review and IPD meta-analysis.	
**Introduction**			
Rationale	3	Describe the rationale for the review in the context of what is already known.	4
Objectives	4	Provide an explicit statement of the questions being addressed with reference, as applicable, to participants, interventions, comparisons, outcomes and study design (PICOS). Include any hypotheses that relate to particular types of participant-level subgroups.	5-6
**Methods**			
Protocol and registration	5	Indicate if a protocol exists and where it can be accessed. If available, provide registration information including registration number and registry name. Provide publication details, if applicable.	Na (PROSPERO registration on page 1)
Eligibility criteria	6	Specify inclusion and exclusion criteria including those relating to participants, interventions, comparisons, outcomes, study design and characteristics (e.g. years when conducted, required minimum follow-up). Note whether these were applied at the study or individual level i.e. whether eligible participants were included (and ineligible participants excluded) from a study that included a wider population than specified by the review inclusion criteria. The rationale for	6

For peer review only

		criteria should be stated.	
Identifying studies - information sources	7	Describe all methods of identifying published and unpublished studies including, as applicable: which bibliographic databases were searched with dates of coverage; details of any hand searching including of conference proceedings; use of study registers and agency or company databases; contact with the original research team and experts in the field; open adverts and surveys. Give the date of last search or elicitation.	6-7
Identifying studies - search	8	Present the full electronic search strategy for at least one database, including any limits used, such that it could be repeated.	Supplementary material
Study selection processes	9	State the process for determining which studies were eligible for inclusion.	7
Data collection processes	10	Describe how IPD were requested, collected and managed, including any processes for querying and confirming data with investigators. If IPD were not sought from any eligible study, the reason for this should be stated (for each such study).	7-9
		If applicable, describe how any studies for which IPD were not available were dealt with. This should include whether, how and what aggregate data were sought or extracted from study reports and publications (such as extracting data independently in duplicate) and any processes for obtaining and confirming these data with investigators.	
Data items	11	Describe how the information and variables to be collected were chosen. List and define all study level and participant level data that were sought, including baseline and follow-up information. If applicable, describe methods of standardising or translating variables within the IPD datasets to ensure common scales or measurements across studies.	7
IPD integrity	A1	Describe what aspects of IPD were subject to data checking (such as sequence generation, data consistency and completeness, baseline imbalance) and how this was done.	8-9
Risk of bias assessment in individual studies.	12	Describe methods used to assess risk of bias in the individual studies and whether this was applied separately for each outcome. If applicable, describe how findings of IPD checking were used to inform the assessment. Report if and how risk of bias assessment was used in any data synthesis.	7
Specification of outcomes and effect measures	13	State all treatment comparisons of interests. State all outcomes addressed and define them in detail. State whether they were pre-specified for the review and, if applicable, whether they were primary/main or secondary/additional outcomes. Give the principal measures of effect (such as risk ratio, hazard ratio, difference in means) used for each outcome.	9-11
Synthesis methods	14	Describe the meta-analysis methods used to synthesise IPD. Specify any statistical methods and models used. Issues should include (but are not restricted to):<br>• Use of a one-stage or two-stage approach.<br>• How effect estimates were generated separately within each study and combined across studies (where applicable).<br>• Specification of one-stage models (where applicable) including how clustering of patients within studies was accounted for.	9-11

For peer review only

		• Use of fixed or random effects models and any other model assumptions, such as proportional hazards.<br>• How (summary) survival curves were generated (where applicable).<br>• Methods for quantifying statistical heterogeneity (such as I^2 and τ^2).<br>• How studies providing IPD and not providing IPD were analysed together (where applicable).<br>• How missing data within the IPD were dealt with (where applicable).	
Exploration of variation in effects	A2	If applicable, describe any methods used to explore variation in effects by study or participant level characteristics (such as estimation of interactions between effect and covariates). State all participant-level characteristics that were analysed as potential effect modifiers, and whether these were pre-specified.	9-11
Risk of bias across studies	15	Specify any assessment of risk of bias relating to the accumulated body of evidence, including any pertaining to not obtaining IPD for particular studies, outcomes or other variables.	7
Additional analyses	16	Describe methods of any additional analyses, including sensitivity analyses. State which of these were pre-specified.	11
**Results**			
Study selection and IPD obtained	17	Give numbers of studies screened, assessed for eligibility, and included in the systematic review with reasons for exclusions at each stage. Indicate the number of studies and participants for which IPD were sought and for which IPD were obtained. For those studies where IPD were not available, give the numbers of studies and participants for which aggregate data were available. Report reasons for non-availability of IPD. Include a flow diagram.	na
Study characteristics	18	For each study, present information on key study and participant characteristics (such as description of interventions, numbers of participants, demographic data, unavailability of outcomes, funding source, and if applicable duration of follow-up). Provide (main) citations for each study. Where applicable, also report similar study characteristics for any studies not providing IPD.	na
IPD integrity	A3	Report any important issues identified in checking IPD or state that there were none.	na
Risk of bias within studies	19	Present data on risk of bias assessments. If applicable, describe whether data checking led to the up-weighting or down-weighting of these assessments. Consider how any potential bias impacts on the robustness of meta-analysis conclusions.	na
Results of individual studies	20	For each comparison and for each main outcome (benefit or harm), for each individual study report the number of eligible participants for which data were obtained and show simple summary data for each intervention group (including, where applicable, the number of events), effect estimates and confidence intervals. These may be tabulated or included on a forest plot.	na
Results of syntheses	21	Present summary effects for each meta-analysis undertaken, including confidence intervals and measures of statistical heterogeneity. State whether the analysis was pre-specified, and report the numbers of studies and participants and, where applicable, the number of events on which it is based.	na

For peer review only

		When exploring variation in effects due to patient or study characteristics, present summary interaction estimates for each characteristic examined, including confidence intervals and measures of statistical heterogeneity. State whether the analysis was pre-specified. State whether any interaction is consistent across trials.	
		Provide a description of the direction and size of effect in terms meaningful to those who would put findings into practice.	
Risk of bias across studies	22	Present results of any assessment of risk of bias relating to the accumulated body of evidence, including any pertaining to the availability and representativeness of available studies, outcomes or other variables.	na
Additional analyses	23	Give results of any additional analyses (e.g. sensitivity analyses). If applicable, this should also include any analyses that incorporate aggregate data for studies that do not have IPD. If applicable, summarise the main meta-analysis results following the inclusion or exclusion of studies for which IPD were not available.	na
**Discussion**			
Summary of evidence	24	Summarise the main findings, including the strength of evidence for each main outcome.	na
Strengths and limitations	25	Discuss any important strengths and limitations of the evidence including the benefits of access to IPD and any limitations arising from IPD that were not available.	na
Conclusions	26	Provide a general interpretation of the findings in the context of other evidence.	na
Implications	A4	Consider relevance to key groups (such as policy makers, service providers and service users). Consider implications for future research.	na
**Funding**			
Funding	27	Describe sources of funding and other support (such as supply of IPD), and the role in the systematic review of those providing such support.	15

A1 – A3 denote new items that are additional to standard PRISMA items. A4 has been created as a result of re-arranging content of the standard PRISMA statement to suit the way that systematic review IPD meta-analyses are reported.

© Reproduced with permission of the PRISMA IPD Group, which encourages sharing and reuse for non-commercial purposes

For peer review only

For peer review only

BMJ Open

The Moderating and Mediating Role of Eating Behaviour Traits in Acceptance and Commitment Therapy-based Weight Management Interventions: Protocol for an Individual Participant Data Meta-analysis

Journal:	*BMJ Open*
Manuscript ID	bmjopen-2023-076411.R1
Article Type:	Protocol
Date Submitted by the Author:	07-Nov-2023
Complete List of Authors:	Kudlek, Laura ; University of Cambridge, MRC Epidemiology Unit<br>Mueller, Julia; University of Cambridge, MRC Epidemiology unit<br>Eustacio Colombo, Patricia ; University of Cambridge, MRC Epidemiology Unit<br>Sharp, Stephen; University of Cambridge, MRC Epidemiology Unit<br>Griffin, Simon; University of Cambridge<br>Ahern, Amy; University of Cambridge, MRC Epidemiology Unit
**Primary Subject Heading**:	Mental health
Secondary Subject Heading:	Diabetes and endocrinology, Epidemiology, Public health
Keywords:	Obesity, NUTRITION & DIETETICS, PREVENTIVE MEDICINE, MENTAL HEALTH

SCHOLARONE™
Manuscripts

The Moderating and Mediating Role of Eating Behaviour Traits in Acceptance and Commitment Therapy-based Weight Management Interventions: Protocol for an Individual Participant Data Meta-analysis

Laura Kudlek*, Julia Mueller, Patricia Eustacio Colombo, Stephen J. Sharp, Simon J Griffin, Amy Ahern

* Corresponding author: laura.kudlek@mrc-epid.cam.ac.uk, MRC Epidemiology Unit, University of Cambridge, UK

For peer review only

1. Abstract

Introduction: Precision medicine approaches to obesity aim to maximise treatment effectiveness by matching weight management interventions to characteristics of individuals, such as eating behaviour traits (EBTs). Acceptance and Commitment Therapy (ACT) - based weight management interventions may address EBTs such as emotional and uncontrolled eating more effectively than standard interventions, and might be most effective in people with high levels of these traits. However, few studies have examined this directly. We will examine (1) whether ACT-based interventions are more effective for people with certain levels of EBTs (i.e. moderation) and (2) whether ACT-based interventions operate through changes in EBTs (i.e. mediation).

Methods and analysis: This Individual Participant Data meta-analysis will follow the Preferred Reporting Items for a Systematic Review and Meta-analysis of Individual Participant Data (PRISMA-IPD) guidance. We will include studies on ACT-based weight management interventions that assessed EBTs in people with a body mass index ≥25kg/m^2. We identified studies by screening studies included in a previous review (Lawlor et al., 2020) of third wave cognitive behavioural interventions, and updating the search to 20.06.2022. We will request individual participant data from eligible published and unpublished studies. We will harmonise and re-analyse data using a two-stage random effects meta-analysis pooling within-trial interactions to investigate moderating effects and using a one-stage simultaneous equation model to examine mediating effects. We will assess the risk of bias of included studies using the Cochrane Risk of Bias tool 2 (RoB2) and the Risk of Bias in Non-randomized Studies of Interventions tool (ROBINS-I).

Ethics and Dissemination: Secondary analysis of pseudonymized data does not require renewed ethical approval. Data sharing will follow data transfer agreements and co-authorship will be offered to investigators contributing data. Findings will be disseminated through peer-reviewed journals and conferences and will contribute to the lead author's PhD thesis.

PROSPERO Registration Number: CRD42022359691

For peer review only

2. Strengths and Limitations

- Obtaining, harmonising and re-analysing individual participant data from systematically identified studies allows us to investigate questions around mediation and moderation that individual studies are typically not powered to detect.
- Individual participant data might not be received from all eligible trials due to unresponsiveness, inability or unwillingness to share data and complex data sharing procedures. This might introduce a possibility for bias.
- Individual studies are likely to exhibit heterogeneity in study populations, nature and intensity of interventions, duration of follow-up, outcome assessment and measures of EBTs, which may challenge the interpretation of data from pooled analyses and necessitate sensitivity analyses.

For peer review only

3. Introduction

Obesity is associated with health risks, such as diabetes, metabolic diseases, and several types of cancer. [1] Worldwide prevalence of overweight and obesity has increased almost threefold since 1975 [1] and is now estimated to be around 60% in the United Kingdom [2] and 70% in the United States. [3] Behavioural weight loss treatments can achieve weight loss, leading to meaningful improvements in health, such as a reduced likelihood of developing diabetes. [4–6] However, outcomes of obesity treatments vary highly among individuals, with some individuals losing significantly more weight than others, and only a proportion of people maintaining weight loss. [7] Precision medicine aims to understand what factors contribute to individual differences in treatment response, and how future intervention content and delivery can be adapted accordingly to maximise their net benefit. [8,9] Eating behaviour traits (EBTs) may be such a factor. [10–14] EBTs are defined as personal tendencies regarding an individual's reaction to food and food-related cues that determine the type, amount, and frequency of food consumed, as well as when to start and stop eating. [15,16] Commonly measured EBTs are restraint (the conscious control of food intake to influence body weight), uncontrolled eating (the tendency to overeat in response to feelings of hunger and external stimuli) and emotional eating (the tendency to overeat in response to negative emotions). [17–19]

During behavioural weight management interventions (WMIs), restraint typically increases and uncontrolled and emotional eating decrease. [20–23] Standard behavioural WMIs involve dietary and physical activity advice, as well as behaviour change techniques such as self-monitoring, goal setting, planning, problem solving, and cognitive restructuring. [24] However, individuals higher in emotional and uncontrolled eating might benefit from additional support targeting these EBTs. [10,25,26] Acceptance and commitment therapy (ACT) aims to increase psychological flexibility (i.e. the capacity to remain in present moment awareness of ones thoughts, feelings, and sensations and accepting these) and to decrease experiential avoidance (i.e. attempts to avoid unpleasant internal experiences). [27] WMIs based on ACT can therefore support the recognition of triggers of overeating as well as the acceptance of negative emotions and cravings, reducing the tendency to rely on food to relieve urges and regulate emotions. ACT-based WMIs are thus theorised to better address emotional and uncontrolled eating compared with standard behavioural techniques. A recent systematic review and meta-analysis concluded that ACT-based WMIs are effective at reducing emotional eating, [28] and a systematic review and network meta-analysis found ACT-based WMIs to be most effective at changing weight outcomes compared to other third wave cognitive behavioural therapies. [29]

However, to determine how useful EBTs and ACT-based WMIs could be for precision medicine approaches, it is also important to understand (a) whether ACT-based WMIs are more effective for people high in emotional and/or uncontrolled eating (i.e., EBTs as effect moderators) and (b) whether changes in EBTs are mechanisms of effectiveness by which ACT-based WMIs impact weight outcomes (i.e., EBTs as effect mediators). Although decreases in emotional and uncontrolled eating have been associated with improved weight loss in a variety of interventions, [12,30–33] few studies

For peer review only

of ACT-based WMIs have examined the role of EBTs in detail. While studies of ACT-based WMIs often measure EBTs, they are mostly reported as outcomes, finding changes in EBTs in the desired direction. [34] Forman et al. additionally explored the moderating role of EBTs, finding ACT-based interventions to be more effective than standard behavioural treatment in people with high initial levels of emotional eating in one study, [35] but not in another. [36] However, sample sizes of these studies were small (N=128 and N=190), and thus likely underpowered for the detection of moderating effects. To our knowledge, no studies of ACT-based interventions have yet examined mediating effects of changes in EBTs.

The scarcity of existing research that has directly examined the mediating and moderating role of EBTs prevents the synthesis of findings in a traditional meta-analysis and makes it difficult to form comprehensive conclusions. Individual Participant Data (IPD) meta-analysis allows us to collate and reanalyse raw data from all relevant studies that have measured the variables of interest, regardless of whether or not the studies have analysed or reported relationships of interest in the original publications. [37] This is likely to increase the number of included studies when compared to a traditional aggregate meta-analysis, and pooling IPD increases the power for analyses that individual studies alone may not be powered to detect. Additionally, heterogeneity is reduced by harmonising data from different measures and re-analysing IPD from original studies according to a uniform analysis plan. [37] Using an IPD meta-analysis thus increases the quantity and quality of data on EBTs in ACT-based WMIs beyond existing evidence, generating a resource to address questions around the moderating and mediating role of EBTs in ACT-based WMIs. This will provide us with new understanding about the potential to use ACT-based interventions to support people with high levels of EBTs as part of a precision medicine approach to obesity treatment that directs specific interventions to those that are most likely to benefit from them.

3.1 Objectives

The aim of this IPD meta-analysis is to obtain and re-analyse IPD from studies assessing EBTs in ACT-based WMIs to examine:

(a) to what extent the effect of ACT-based WMIs on weight loss depends on individuals' levels of EBTs
(b) to what extent changes in EBTs mediate the effect of ACT-based WMIs on weight loss

4. Methods and analysis

This project will follow guidance from the Preferred Reporting Items for Systematic Reviews and Meta-Analyses for IPD extension (PRISMA-IPD).[38] The protocol will additionally follow the PRISMA protocol extension (PRISMA-P) where applicable. The phases of planning and conducting an IPD meta-analysis differ from those of standard aggregate systematic reviews and meta-analyses, with

For peer review only - http://bmjopen.bmj.com/site/about/guidelines.xhtml

some occurring concurrently, [39] We will thus communicate the current stage of work on the project by using either past or future tense to describe relevant sections.

4.1 Study identification

4.1.1 Eligibility criteria

Data from published and unpublished studies were considered eligible for inclusion if studies met the following criteria:

- **Population:** Adults (aged 18 and older) with a BMI ≥ 25 kg/m^2. Studies were excluded if participants were recruited purely based on having a chronic disease or being pregnant, as were studies where eligible participants resided in institutional settings (e.g. hospital, army barracks). Studies on children and adolescents were not considered for inclusion to avoid the risk of increasing heterogeneity in interventions serving different target populations.
- **Intervention:** Interventions with the primary goal of weight loss or weight loss maintenance (referred to as weight management interventions in the remainder of this protocol) that report incorporating strategies based on ACT. ACT-based interventions from different contexts were eligible (e.g. online, in person, health care setting, commercial), and they were eligible either as standalone treatment or as part of a wider weight management intervention.
- **Comparators:** No/ wait-list control, minimal intervention (e.g. leaflet, brief advice), or standard behavioural WMI. Studies with no control group will be eligible for a subset of analyses.
- **Outcome:** Weight assessed at post-treatment or both at post-treatment and any follow-up point. A follow-up point of at least 3-months post-baseline had to be available.
- **Mediators/ Moderators:** EBTs assessed at baseline, post-treatment or both. Eligible EBTs are emotional eating, uncontrolled eating, disinhibition, external eating and restraint. Disordered eating will be excluded.
- **Study design:** Due to the scarcity of research in the field, we will include all types of intervention studies to obtain as much IPD as possible (randomised controlled trials (RCTs), non-RCTs, prospective cohort and case series studies). However, only RCTs and cluster-RCTs will be eligible for the full set of analyses.

4.1.2 Search strategy

We screened studies included in a review by Lawlor et al. (2020) [29] on third wave cognitive behavioural therapies for weight management against this review's eligibility criteria. In addition, we re-ran an adapted search relating to the concepts of (a) ACT and (b) overweight, obesity or weight management from the 25th of September 2019 until the 20th of June 2022 (see Supplementary Material). Concepts relating to other third wave cognitive behavioural therapies were deleted from the

For peer review only - http://bmjopen.bmj.com/site/about/guidelines.xhtml

original search strategy to match the narrower focus of this IPD meta-analysis. No restrictions on language were applied. We searched the databases MEDLINE, CINAHL, Embase, PsycINFO, AMED, ASSIA, Web of Science, the Cochrane database (CENTRAL) and hand-searched the reference lists of key publications. Furthermore, when requesting IPD, all authors of included studies will be asked whether they are aware of any additional eligible published or unpublished data.

4.1.3 Study selection

Two independent reviewers, PEC and LK, screened both title and abstracts and full texts in Covidence. [40] Disagreements were resolved by discussion and consulting of a third reviewer, AA, where necessary. We will contact authors of protocols and conference abstracts to resolve any outstanding questions on eligibility and to enquire about their willingness to share unpublished results.

4.2 Risk of bias assessment

The Cochrane Risk of Bias tool 2 (RoB2) [41] or the Risk of Bias in Non-randomised Studies of Interventions tool (ROBINS-I) [42] will be used by two researchers independently to assess risk of bias. Disagreements will be resolved by discussion and consulting of a third reviewer if necessary.

4.3 Variables requested

The following variables will be requested in a detailed data dictionary, including definitions.

- De-identified participant ID
- Age at Baseline
- Sex
- Height at baseline
- Weight at baseline, end of intervention and any follow-up
- Allocated trial arm
- Number of sessions attended
- All measured EBTs at baseline, end of intervention and any follow up point
- All measures of experiential avoidance and/or psychological flexibility at baseline, end of intervention, and any follow-up point
- Variables that describe which participants were excluded from main analysis and why

For peer review only

4.4 Data collection and management

4.4.1 Extraction of published data on study characteristics

Data on study characteristics will be extracted from published manuscripts by two reviewers independently, using a form that will be adapted from the Cochrane data extraction form (Supplementary Material). [43] These include data on study design and setting, participant sociodemographic characteristics (e.g. ethnicity, education, socioeconomic status) and details on the intervention and control conditions. A third reviewer will cross-check extractions for any discrepancies, and original study authors will be provided with an opportunity to cross-check extractions for accuracy, including any potential updates.

4.4.2 Requesting IPD

Authors will be contacted via email, outlining the purpose of the study, providing the study protocol, and asking them to collaborate on the project by providing IPD. If the corresponding authors agree, more detailed instructions including an outline of the specific data requested and a data dictionary will be shared. If authors are not able to share their data, we will ask them to perform the analyses using a pre-specified protocol and share the results so that we can include them in the meta-analysis. Authors will be sent two reminders after initial contact, each with a time-period of around three weeks between them. If no response is given and published results are not suitable for meta-analysis, the study concerned will be excluded. Studies that are excluded due to inability to contact will be summarised in a table in the supplementary materials of the publication.

4.4.3 Collecting IPD

Data will be accepted in any format, but a Microsoft Excel format is preferred. A data dictionary will be provided prior to data transfer. Any data sharing will strictly follow the conditions pre-specified in data transfer agreements, and all data will be shared via, and stored in, the MRC Epidemiology Unit's secure research drive. After receiving data, a copy will be saved that will be kept as original. Working files will be converted and imported into R version 4.1.2. [44]

4.4.4 Data harmonisation

Data will be harmonised according to the pre-specified data dictionary to merge it into a combined dataset. Variables will be recoded and transformed using a uniform coding scheme (e.g. height and weight will be transformed into metric units, age will be converted into age in years, etc.). EBTs are likely to have been assessed using different questionnaires across studies (e.g. TFEQ-R18, TFEQ-51, DEBQ, etc.). To harmonise EBT data, we will ensure that all EBT outcome scores represent the

For peer review only - http://bmjopen.bmj.com/site/about/guidelines.xhtml

relative proportion of highest possible raw scores on a 0 to 100 range. To convert EBT outcome scores that do not follow this scoring approach, we will subtract the raw outcome score by the lowest possible raw score, divide this by the possible score range and multiply this by 100. To facilitate this, we will use item-level data where available.

4.4.5 Data checking

Data received will be compared to that reported in the original publication by running descriptive statistics (e.g. sample size, weight loss outcomes). Additionally, if both item and subscale-levels of EBTs are provided, subscales will be re-computed and compared to those provided to confirm item scoring and ensure the uniform allocation of items to subscales. In case of any discrepancies, authors will be contacted for clarification. If discrepancies cannot be resolved, the nature and severity of the deviation will be evaluated to determine whether the study can be included. After a study has been checked, a summary will be sent to the original authors to give them the opportunity to address any issues before merging the study into the pooled dataset.

4.4.6 Database creation and aggregation

A merged dataset containing harmonised data from all included studies will be created for convenience when managing and analysing data. Data from different studies will be distinguished using a unique identifier for each study that is allocated before the final merging. To check for any errors introduced during the merging phase, descriptive statistics of each study will be run before merging datasets and compared to corresponding statistics produced after merging.

4.4.7 Studies where IPD is not available

If authors are unable to share their raw data, we will ask them to perform the required analyses for us and share their results (i.e. a federated approach). This will allow us to still include the study in the moderation analyses. To check whether this might introduce bias, we will conduct sensitivity analysis using only the data from studies providing IPD. If authors can neither provide IPD, nor provide the outcome statistics of requested analyses (i.e. federated data), any published data will be synthesised and compared to included studies. This will include available published data on sociodemographic variables, such as socioeconomic status, sex, and age, as well as any reported data on eating behaviours at baseline and end of intervention, and weight data at baseline, end of intervention and follow-up. As with data on study characteristics, published data on EBTs and weight outcomes will be extracted in duplicate using a pre-specified data extraction form based on the Cochrane data extraction form template. [43]

For peer review only

4.5 Data analyses

4.5.1 Descriptive statistics

Descriptive statistics and the proportion of missing values of age, sex, baseline EBTs and baseline BMI will be derived from the raw data directly. These will be reported for each study, as well as for the overall sample included in the meta-analyses. Other study characteristics, of which IPD will not be requested, e.g. SES and ethnicity, will be extracted from published reports.

4.5.2 Statistical analysis

To gain a more complete picture of the role of EBTs in WMIs, we will explore to what extent (1) baseline EBTs are associated with changes in weight over the intervention and follow-up period, and (2) to what extent changes in EBTs are associated with changes in weight over the intervention and follow-up period before conducting the main moderation and mediation analyses. These initial associations will be explored using linear regression, adjusted for study arm, age, sex, baseline weight and duration of follow up. This will allow us to incorporate evidence from studies without a control group. Findings will be synthesised using a two-stage IPD meta-analysis approach, [37] in which analyses are first performed for each study individually, and then the results are combined using meta-analysis. This allows for the incorporation of studies that do not provide IPD if collaborators perform requested analyses at their home institutions and share only their output. We will use random-effects meta-analyses to accommodate for heterogeneity between studies.

4.5.2.1 Moderation

To investigate whether the effect of ACT-based interventions on weight depends on people's levels of emotional and uncontrolled eating, we will use a two-stage IPD-MA approach and perform a random-effects meta-analysis to combine within-trial regression coefficients of the intervention*EBT interactions. To facilitate interpretation of potential interaction effects with EBT as a continuous variable, we will also fit regression models in three EBT subgroups (reflecting "low", "medium", and "high" levels of the respective EBT) in each trial and combine the estimated subgroup-specific effects using random-effects meta-analysis. Subgroups will be determined by using the tertile EBT scores of the pooled sample as cut-offs for each individual trial. We will investigate the potential moderating effects of EBTs on weight loss outcomes at the end of the intervention as well as at available follow-up timepoints (e.g. 6 and 12 months).

For peer review only - http://bmjopen.bmj.com/site/about/guidelines.xhtml

4.5.2.2 Mediation

Mediation analyses will follow ‘A Guideline for Reporting Mediation Analyses’ (AGReMA) guidelines. [45] We will use the simultaneous equations model (SEM) approach outlined by Huh et al. 2022 [46] to estimate (a) the direct effect by which ACT-based interventions impact weight loss, (b) the indirect effect by which they impact weight loss via changes in EBTs, (c) and the total effect by which weight loss is impacted. We will estimate direct, indirect and total effects on weight loss outcomes at the end of the intervention as well as at available follow-up timepoints (e.g. 6 and 12 months). Figure 1 and figure 2 depict the path diagrams that will be tested both in each individual trial, i.e. “study-specific sub-models”, and in a pooled sample using a one-stage approach, i.e. “overall model”. The SEM for the overall models will control for clustering within studies by using complex survey analysis. While the one-stage approach eliminates the risk of aggregation/ ecological bias, it requires IPD, making a federated approach (where authors share only their output) impossible. Thus, studies that cannot provide IPD will be excluded for this part of the analysis. If trials with less than 50 participants per intervention arm will be included, we will consider adapting the path diagram depicted in Figures 1 and 2 by adding the variables age and height with a path to both the outcome and intervention arm to control for possible imbalances between the randomised groups. If it is concluded from previous moderation analyses (Section 4.5.2.1) that ACT-based interventions only have a significant effect on weight outcomes in one or two of the three EBT level subgroups (low, medium or high EBT levels), then we will repeat the mediation analyses in the subgroup(s) that did show a significant effect on weight outcomes. Analyses will be performed in R, [44] using the lavaan [47] and lavaan.survey [48] packages.

4.5.3 Sensitivity Analyses

We will conduct a series of sensitivity analyses. At the study-level, we will compare results from analyses performed in (a) the full set of studies versus excluding studies classified at high risk of bias using the RoB2 (also see section *4.2 Risk of Bias Assessment*) (b) studies providing IPD data versus studies providing federated data only (also see section *4.4.7 Studies where IPD is not available*) (c) studies with waitlist or minimal control conditions versus studies with standard behavioural control conditions (d) studies that significantly reduced experiential avoidance (as this is the hypothesised mechanism of action of ACT-based WMIs) versus studies that did not significantly reduce experiential avoidance. At the individual level, we will compare results from analyses performed in (e) all individuals versus those that received a sufficient dose of the intervention (as determined from liaising with study authors). Details will be described in a separate analysis plan that will be shared via the Open Science Framework (OSF). [49]

For peer review only

4.6 Patient and Public Involvement (PPI)

We will seek PPI input for the interpretation of analyses and implication of results in the form of remote focus group meetings with two or more PPI members. This may result in co-authorship on the publication.

5. Ethics and Dissemination

This IPD meta-analysis protocol is pre-registered (PROSPERO: CRD42022359691). The collation and secondary analysis of pseudonymized IPD does not require new ethical approval. Data from individual trials will be shared with the lead institution under appropriate data sharing agreements. We will disseminate findings through peer-reviewed journals and conferences. Investigators contributing data to this analysis will be offered co-authorship. This study will contribute to the lead author's PhD thesis.

For peer review only

6. Authors Contributions

LK conceived and designed study plans, developed the analysis strategy, performed initial screening and drafted the protocol manuscript. AA provided input on the conception and development of study plans, including the analysis strategy, contributed to screening, and reviewed drafts of the manuscript. JM contributed to the development of study plans, in particular the analysis strategy, and reviewed drafts of the manuscript. SJS provided input on the analysis strategy and reviewed drafts of the manuscript. PEC provided input on study design, performed initial screening and reviewed drafts of the manuscript. SJG provided input on study design and reviewed drafts of the manuscript. All authors have read and approved the final version of the manuscript.

7. Data Availability Statement

This IPD meta-analysis will use data that is obtained under data sharing contracts with the owners of individual data sets. Since these contracts do not allow for onward sharing, requests for IPD should be made to the original owners of the data.

8. Funding Statement

This work was supported by the Medical Research Council MC_UU_00006/6 as part of LK's PhD

For peer review only - http://bmjopen.bmj.com/site/about/guidelines.xhtml

9. Competing Interest Statement

AA, JM, SJS and SJG were authors of two studies identified as eligible for this IPD meta-analysis (SWiM-F and SWiM-C). JM is a Trustee for the Association for the Study of Obesity (unpaid role). SG has received honoraria from Astra Zeneca for contributing to postgraduate education sessions for primary care teams concerning the management of type 2 diabetes. SG is a trustee of the Novo Nordisk UK Research Foundation. AA is a member of the WW Scientific Advisory Board.

For peer review only

For peer review only - http://bmjopen.bmj.com/site/about/guidelines.xhtml

10. References

1 World Health Organization. Obesity and overweight. 2021.

2 NHS digital. Health survey for England 2019. 2020;1–22.

3 National Institute of Diabetes and Digestive and Kidney Disease. Overweight & Obesity Statistics. 2021. https://www.niddk.nih.gov/health-information/health-statistics/overweight-obesity

4 Twells LK, Harris Walsh K, Blackmore A, *et al.* Nonsurgical weight loss interventions: A systematic review of systematic reviews and meta-analyses. *Obesity Reviews*. 2021;22. doi: 10.1111/obr.13320

5 Hartmann-Boyce J, Johns DJ, Jebb SA, *et al.* Behavioural weight management programmes for adults assessed by trials conducted in everyday contexts: Systematic review and meta-analysis. *Obesity Reviews*. 2014;15:920–32.

6 LeBlanc ES, Patnode CD, Webber EM, *et al.* Behavioral and pharmacotherapy weight loss interventions to prevent obesity-related morbidity and mortality in adults updated evidence report and systematic review for the US preventive services task force. *JAMA - Journal of the American Medical Association*. 2018;320:1172–91.

7 MacLean PS, Wing RR, Davidson T, *et al.* NIH working group report: Innovative research to improve maintenance of weight loss. *Obesity*. 2015;23:7–15.

8 U. S. Food and Drug Administration (FDA). Precision Medicine. 2018. https://www.fda.gov/medical-devices/in-vitro-diagnostics/precision-medicine (accessed 5 July 2022)

9 Bomberg EM, Ryder JR, Brundage RC, *et al.* Precision medicine in adult and pediatric obesity: a clinical perspective. Ther Adv Endocrinol Metab. 2019;10. https://doi.org/10.1177/2042018819863022

10 van Strien T. Causes of Emotional Eating and Matched Treatment of Obesity. Curr Diab Rep. 2018;18. https://doi.org/10.1007/s11892-018-1000-x

11 Boutelle KN, Manzano MA, Eichen DM. Appetitive traits as targets for weight loss: The role of food cue responsiveness and satiety responsiveness. *Physiol Behav*. 2020;224:113018.

12 Braden A, Flatt SW, Boutelle KN, *et al.* Emotional eating is associated with weight loss success among adults enrolled in a weight loss program. *J Behav Med*. 2016;39:727–32.

13 Boutelle KN, Eichen DM, Peterson CB, *et al.* Effect of a Novel Intervention Targeting Appetitive Traits on Body Mass Index Among Adults With Overweight or Obesity. *JAMA Netw Open*. 2022;5:e2212354.

14 Niemeier HM, Leahey T, Palm Reed K, *et al.* An Acceptance-Based Behavioral Intervention for Weight Loss: A Pilot Study. *Behav Ther*. 2012;43:427–35.

15 Llewellyn, Fildes A. Behavioural Susceptibility Theory: Professor Jane Wardle and the Role of Appetite in Genetic Risk of Obesity. *Curr Obes Rep*. 2017;6:38–45.

16 Llewellyn C, Wardle J. Behavioral susceptibility to obesity: Gene–environment interplay in the development of weight. *Physiol Behav*. 2015;152:494–501.

17 Stunkard AJ, Messick S. Eating Inventory. *APA PsychTests*. 1988.

For peer review only

18 Karlsson J, Persson L-O, Sjo L, *et al.* Psychometric properties and factor structure of the Three-Factor Eating Questionnaire (TFEQ) in obese men and women. Results from the Swedish Obese Subjects (SOS) study. 2000. www.nature.com/ijo

19 Tholin S, Rasmussen F, Tynelius P, *et al.* Genetic and environmental influences on eating behavior: the Swedish Young Male Twins Study. *American Journal of Clinical Nutrition*. 2005.

20 Provencher V, Bégin C, Tremblay A, *et al.* Health-At-Every-Size and Eating Behaviors: 1-Year Follow-Up Results of a Size Acceptance Intervention. *J Am Diet Assoc*. 2009;109:1854–61.

21 Nurkkala M, Kaikkonen K, Vanhala ML, *et al.* Lifestyle intervention has a beneficial effect on eating behavior and long-term weight loss in obese adults. *Eat Behav*. 2015;18:179–85.

22 Chew HSJ, Lau ST, Lau Y. Weight-loss interventions for improving emotional eating among adults with high body mass index: A systematic review with meta-analysis and meta-regression. *European Eating Disorders Review*. Published Online First: 23 April 2022. doi: 10.1002/erv.2906

23 Mason C, de Dieu Tapsoba J, Duggan C, *et al.* Eating behaviors and weight loss outcomes in a 12-month randomized trial of diet and/or exercise intervention in postmenopausal women. *International Journal of Behavioral Nutrition and Physical Activity*. 2019;16. doi: 10.1186/s12966-019-0887-1

24 National Institute for Health and Care Excellence. Obesity: identification, assessment and management. https://www.nice.org.uk/guidance/cg189/chapter/1-Recommendations#behavioural-interventions. 2014.

25 Lillis J, Niemeier HM, Ross KM, *et al.* Weight loss intervention for individuals with high internal disinhibition: Design of the Acceptance Based Behavioral Intervention (ABBI) randomized controlled trial. *BMC Psychol*. 2015;3:1–10.

26 Braden A, Ph D, Flatt SW, *et al.* Emotional Eating is Associated with Weight Loss Success among Adults Enrolled in a Weight Loss Program. *J Behav Med*. 2016;39:727–32.

27 Hayes SC. Acceptance and Commitment Therapy, Relational Frame Theory, and the Third Wave of Behavioral and Cognitive Therapies. *Behav Ther*. 2004;35:639–65.

28 Di Sante J, Akeson B, Gossack A, *et al.* Efficacy of ACT-based treatments for dysregulated eating behaviours: A systematic review and meta-analysis. Appetite. 2022;171. https://doi.org/10.1016/j.appet.2022.105929

29 Lawlor ER, Islam N, Bates S, *et al.* Third-wave cognitive behaviour therapies for weight management: A systematic review and network meta-analysis. *Obesity Reviews*. 2020;21:1–21.

30 Keränen AM, Savolainen MJ, Reponen AH, *et al.* The effect of eating behavior on weight loss and maintenance during a lifestyle intervention. *Prev Med (Baltim)*. 2009;49:32–8.

31 Butryn ML, Thomas JG, Lowe MR. Reductions in internal disinhibition during weight loss predict better weight loss maintenance. *Obesity (Silver Spring)*. 2009;17:1101–3.

32 Nurkkala M, Kaikkonen K, Vanhala ML, *et al.* Lifestyle intervention has a beneficial effect on eating behavior and long-term weight loss in obese adults. *Eat Behav*. 2015;18:179–85.

33 Urbanek JK, Metzgar CJ, Hsiao PY, *et al.* Increase in cognitive eating restraint predicts weight loss and change in other anthropometric measurements in overweight/obese premenopausal women. *Appetite*. 2015;87:244–50.

34 Mueller J, Richards R, Jones RA, *et al.* Supporting Weight Management during COVID-19 (SWiM-C): A randomised controlled trial of a web-based, ACT-based, guided self-help intervention. *Obes Facts*. Published Online First: 13 April 2022. doi: 10.1159/000524031

For peer review only

For peer review only - http://bmjopen.bmj.com/site/about/guidelines.xhtml

35 Forman EM, Butryn ML, Juarascio AS, *et al.* The mind your health project: A randomized controlled trial of an innovative behavioral treatment for obesity. *Obesity*. 2013;21:1119–26.

36 Forman EM, Butryn ML, Manasse SM, *et al.* Acceptance-based versus standard behavioral treatment for obesity: Results from the mind your health randomized controlled trial. *Obesity*. 2016;24:2050–6.

37 Riley RD, Stewart LA, Tierney JF. *Individual Participant Data Meta-Analysis for Healthcare Research*. Wiley 2021. https://doi.org/10.1002/9781119333784.ch1

38 Stewart LA, Clarke M, Rovers M, *et al.* Preferred reporting items for a systematic review and meta-analysis of individual participant data: The PRISMA-IPD statement. *JAMA - Journal of the American Medical Association*. 2015;313:1657–65.

39 Stewart LA, Riley RD, Tierney JF. Planning and Initiating and IPD Meta-Analysis Project. In: Riley RD, Tierney JF, Stewart LA, eds. *Individual Participant Data Meta-Analysis: A Handbook for healthcare Research*. John Wiley & Sons Ltd. 2021:22–4.

40 Veritas Health Innovation. Covidence systematic review software. 2022. www.covidence.org (accessed 6 July 2022)

41 Higgins J, Savović J, Page M, *et al.* Chapter 8: Assessing risk of bias in a randomized trial. In: Higgins J, Thomas J, Chandler J, *et al.*, eds. *Cochrane Handbook for Systematic Reviews of Interventions*. Cochrane 2022. www.training.cochrane.org/handbook (accessed 23 June 2022)

42 Sterne JA, Hernán MA, Reeves BC, *et al.* ROBINS-I: a tool for assessing risk of bias in non-randomised studies of interventions. *BMJ*. 2016;i4919.

43 Cochrane Developmental Psychosocial and Learning Problems. Data Extraction Forms. 2014. https://dplp.cochrane.org/data-extraction-forms (accessed 23 June 2022)

44 R Core Team. R: A language and environment for statistical computing. 2021. https://www.R-project.org/ (accessed 23 June 2022)

45 Lee H, Cashin AG, Lamb SE, *et al.* A Guideline for Reporting Mediation Analyses of Randomized Trials and Observational Studies: The AGReMA Statement. *JAMA - Journal of the American Medical Association*. 2021;326:1045–56.

46 Huh D, Li X, Zhou Z, *et al.* A Structural Equation Modeling Approach to Meta-analytic Mediation Analysis Using Individual Participant Data: Testing Protective Behavioral Strategies as a Mediator of Brief Motivational Intervention Effects on Alcohol-Related Problems. *Prevention Science*. 2022;23:390–402.

47 Rosseel Y. Journal of Statistical Software lavaan: An R Package for Structural Equation Modeling. 2012. http://www.jstatsoft.org/

48 Oberski D. lavaan.survey: An R Package for Complex Survey Analysis of Structural Equation Models. 2014. http://www.jstatsoft.org/

49 Center for Open Science. Open Science Framework (OSF). https://osf.io (accessed 13 October 2023)

For peer review only

11. Figure legends

Figure 1: Path diagram for individual EBTs

Figure 2: Joint path diagram combining individual EBTs (e.g. emotional eating (EE), uncontrolled eating (UE) and restraint (R))

For peer review only

For peer review only - http://bmjopen.bmj.com/site/about/guidelines.xhtml

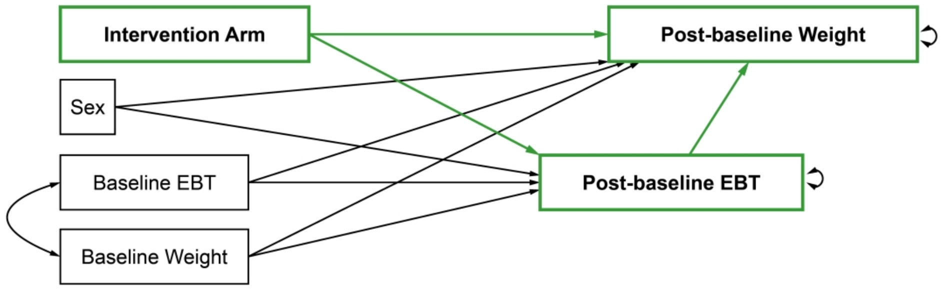

Figure 1: Path diagram for individual EBTs

397x124mm (300 x 300 DPI)

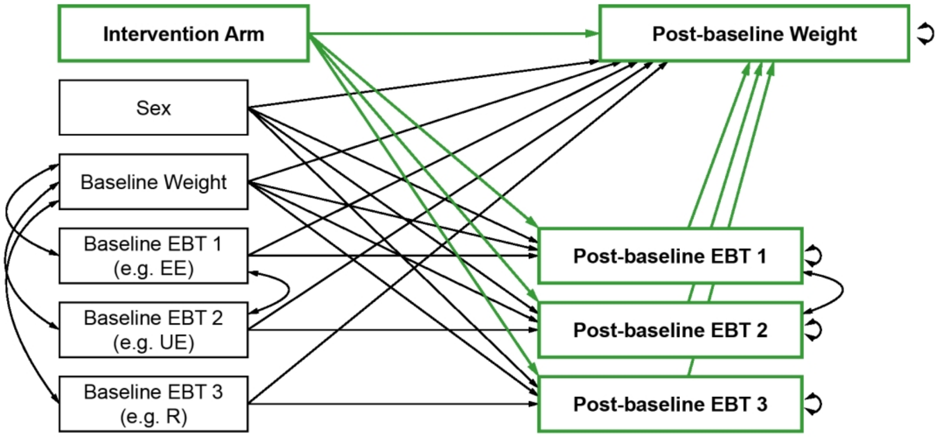

Figure 2: Joint path diagram combining individual EBTs (e.g. emotional eating (EE), uncontrolled eating (UE) and restraint (R))

397x188mm (118 x 118 DPI)

The Moderating and Mediating Role of Eating Behaviour Traits in Acceptance and Commitment Therapy-based Weight Management Interventions: Protocol for an Individual Participant Data Meta-analysis

[SUPPLEMENTARY MATERIAL]

Laura Kudlek [a]*, Julia Mueller [a], Patricia Eustacio Colombo [a], Stephen J. Sharp [a] , Simon J Griffin [a, b], Amy Ahern [a]

[a] MRC Epidemiology Unit, School of Clinical Medicine, University of Cambridge, Cambridge, United Kingdom

[b] Primary Care Unit, Department of Public Health and Primary Care, University of Cambridge, Cambridge, United Kingdom

* Corresponding author, email: laura.kudlek@mrc-epid.cam.ac.uk

For peer review only

Contents

1.0 SEARCH STRATEGY 3

1.1 MEDLINE via Ovid 3

1.2 EMBASE via Ovid 3

1.3 AMED via Ovid 3

1.4 CENTRAL via Cochrane 4

1.5 ASSIA via ProQuest 4

1.6 PSYCINFO via ProQuest 5

1.7 WEB OF SCIENCE via web of science 5

1.8 CINAHL via EBSCOhost 5

2.0 DATA EXTRACTION FORM 6

2.1 Top-line details 6

2.2 Study characteristics 6

2.2.1 Methods 6
2.2.2 Notes 6
2.2.3 Participants (at baseline) 7
2.2.4 Interventions 7

2.3 Mediators/Moderators/Outcomes 8

For peer review only

1.0 Search Strategy

We screened studies included in a review by Lawlor et al. (2020) [28] on third wave cognitive behavioural therapies for weight management against this review's eligibility criteria. In addition, we re-ran an adapted search relating to the concepts of (a) ACT and (b) overweight, obesity or weight management from the 25th of September 2019 until the 20th of June 2022. Details of the adapted search strategy are shown below.

1.1 MEDLINE via Ovid

**1**	exp Obesity/ OR exp Overweight/ OR exp Body Weight/ OR exp Body Mass Index/ OR exp Waist Circumference/ OR exp Feeding Behavior/ OR exp Body Weight Changes/ OR exp Caloric Restriction/ OR exp Weight Loss/ OR obes*.mp OR (overweight or over-weight).mp OR (weight adj3 (body or chang* or loss* or maint* or manag* or control* or reduct*)).mp OR (food adj3 (intake or habit*)).mp OR (body mass index or bmi).mp OR body adj3 mass.mp OR (calori* adj3 (restrict* or restrain* or reduc*)).mp OR feeding adj3 behavio*.mp OR (diet* adj3 (restrict* or restrain* or reduc*)).mp OR (waist* adj3 circumferenc*).mp
**2**	"Acceptance and Commitment Therapy"/ OR (acceptance* adj3 (commit* or mind* or base* or focus* or intervention* or therap* or treat*)).mp
**3**	1 AND 2
**4**	Limit 3 to dt=20190925-20220620
**5**	Limit 3 to rd=20190925-20220620
**6**	4 OR 5

1.2 EMBASE via Ovid

**1**	exp Obesity/ OR exp Body Weight/ OR exp Body Mass/ OR exp Waist Circumference/ OR exp Feeding Behavior/ OR exp Caloric Restriction/ OR exp Weight Reduction/ OR obes*.mp OR (overweight or over-weight).mp OR (weight adj3 (body or chang* or loss* or maint* or manag* or control* or reduct*)).mp OR (food adj3 (intake or habit*)).mp OR (body mass index or bmi).mp OR (body adj3 mass).mp OR (calori* adj3 (restrict* or restrain* or reduc*)).mp OR (feeding adj3 behavio*).mp OR (diet* adj3 (restrict* or restrain* or reduc*)).mp OR (waist* adj3 circumferenc*).mp
**2**	"acceptance and commitment therapy"/ OR (acceptance* adj3 (commit* or mind* or base* or focus* or intervention* or therap* or treat*)).mp
**3**	1 AND 2
**4**	Limit 3 to dd=20190925-20220620
**5**	Limit 3 to rd=20190925-20220620
**6**	4 OR 5

1.3 AMED via Ovid

**1**	exp Obesity/ or exp Body Weight/ or exp Body Mass Index/ or exp Weight Loss/ or obes*.mp or (overweight or over-weight).mp or (weight adj3 (body or chang* or loss* or maint* or manag* or control* or reduct*)).mp or (food adj3 (intake or habit*)).mp or (body mass index or bmi).mp or body adj3 mass.mp or (calori* adj3 (restrict* or restrain* or reduc*)).mp or feeding adj3 behavio*.mp or (diet* adj3 (restrict* or restrain* or reduc*)).mp or (waist* adj3 circumferenc*).mp

For peer review only

**2**	(acceptance* adj3 (commit* or mind* or base* or focus* or intervention* or therap* or treat*)).mp
**3**	1 AND 2
**4**	Limit 3 to 2018-Current

1.4 CENTRAL via Cochrane

**#1**	MeSH descriptor: [Obesity] explode all trees	15762
**#2**	MeSH descriptor: [Overweight] explode all trees	18878
**#3**	MeSH descriptor: [Body Weight] explode all trees	31138
**#4**	MeSH descriptor: [Body Mass Index] explode all trees	10927
**#5**	MeSH descriptor: [Waist Circumference] explode all trees	1143
**#6**	MeSH descriptor: [Feeding Behavior] explode all trees	9769
**#7**	MeSH descriptor: [Body Weight Changes] explode all trees	9695
**#8**	MeSH descriptor: [Caloric Restriction] explode all trees	941
**#9**	MeSH descriptor: [Weight Loss] explode all trees	7104
**#10**	obes* in All Text	51603
**#11**	(overweight or over-weight)	19265
**#12**	(weight near/3 (body or chang* or loss* or maint* or manag* or control* or reduct*))	75571
**#13**	(food near/3 (intake or habit*))	11473
**#14**	(body mass index or bmi)	74353
**#15**	(body near/3 mass)	64975
**#16**	(calori* near/3 (restrict* or restrain* or reduc*))	3305
**#17**	feeding near/3 behavio*	5662
**#18**	(diet* near/3 (restrict* or restrain* or reduc*))	15513
**#19**	(waist* near/3 circumferenc*)	10789
**#20**	#1 or #2 or #3 or #4 or #5 or #6 or #7 or #8 or #9 or #10 or #11 or #12 or #13 or #14 or #15 or #16 or #17 or #18 or #19	171531
**#21**	MeSH descriptor: [Acceptance and Commitment Therapy] explode all trees	281
**#22**	(acceptance* near/3 (commit* or mind* or base* or focus* or intervention* or therap* or treat*))	2898
**#23**	#21 or #22	2898
**#24**	#20 and #23	324
	(with Cochrane Library publication date from Aug 2019 to Jun 2022) Apply limits > Select limits > Run search	132

1.5 ASSIA via ProQuest

MAINSUBJECT.EXACT.EXPLODE("Obesity") OR MAINSUBJECT.EXACT.EXPLODE("Body weight") OR MAINSUBJECT.EXACT.EXPLODE("Body Mass Index") OR

MAINSUBJECT.EXACT.EXPLODE("Feeding patterns") OR MAINSUBJECT.EXACT.EXPLODE("Caloric intake") OR obes* OR overweight OR over-weight OR (weight NEAR/3 (body or chang* or loss* or maint* or manag* or control* or reduct*)) OR (food NEAR/3 (intake or habit*)) OR (“body mass index” or bmi) OR (body NEAR/3 mass) OR (calori* NEAR/3 (restrict* or restrain* or reduc*)) OR (feeding NEAR/3 (pattern* or behavio*)) OR (diet* NEAR/3 (restrict* or restrain* or reduc*)) OR (waist* NEAR/3 circumferenc*)
AND
acceptance* NEAR/3 (commit* or mind* or base* or focus* or intervention* or therap* or treat*)
Limit by publication date (2019-09-25 to 2022-06-21)

1.6 PSYCINFO via ProQuest

MAINSUBJECT.EXACT.EXPLODE("Obesity") OR MAINSUBJECT.EXACT.EXPLODE("Body Weight") OR MAINSUBJECT.EXACT.EXPLODE("Body Mass Index") OR MAINSUBJECT.EXACT.EXPLODE("Eating Behavior") OR MAINSUBJECT.EXACT.EXPLODE("Food Intake") OR MAINSUBJECT.EXACT.EXPLODE("Diets") OR obes* OR overweight OR over-weight OR (weight NEAR/3 (body or chang* or loss* or maint* or manag* or control* or reduct*)) OR (food NEAR/3 (intake or habit*)) OR (“body mass index” or bmi) OR (body NEAR/3 mass) OR (calori* NEAR/3 (restrict* or restrain* or reduc*)) OR (feeding NEAR/3 (pattern* or behavio*)) OR (diet* NEAR/3 (restrict* or restrain* or reduc*)) OR (waist* NEAR/3 circumferenc*)
AND
MAINSUBJECT.EXACT.EXPLODE(“Acceptance and Commitment Therapy”) OR acceptance* NEAR/3 (commit* or mind* or base* or focus* or intervention* or therap* or treat*)
Filtered by 2019-09-25 - 2022-06-21

1.7 WEB OF SCIENCE via web of science

(TS=(obes*) OR TS=(overweight or over-weight) OR TS=(weight NEAR/3 (body or chang* or loss* or maint* or manag* or control* or reduct*)) OR TS=(food NEAR/3 (intake or habit*)) OR TS=(“body mass index” or bmi) OR TS=(body NEAR/3 mass) OR TS=(calori* NEAR/3 (restrict* or restrain* or reduc*)) OR TS=(feeding NEAR/3 behavio*) OR TS=(diet* NEAR/3 (restrict* or restrain* or reduc*)) OR TS=(waist* NEAR/3 circumferenc*))
AND
(TS=(acceptance* NEAR/3 (commit* or mind* or base* or focus* or intervention* or therap* or treat*)))
Refined by: PUBLICATION date (2019-09-25 to 2022-06-20)

1.8 CINAHL via EBSCOhost

((MH "Obesity+") OR (MH "Body Weight+") OR (MH "Eating Behavior+") OR (MH "Body Weight Changes+") OR (MH "Weight Loss+") OR (MH "Body Mass Index") OR (MH "Waist Circumference") OR (MH "Weight Reduction Programs") OR (TX obes*) OR (TX (overweight or over-weight)) OR (TX (weight N3 (body or chang* or loss* or maint* or manag* or control* or reduct*))) OR (TX (food N3 (intake or habit*))) OR (TX (body mass index or bmi)) OR (TX (body N3 mass)) OR (TX (calori* N3 (restrict* or restrain* or reduc*))) OR (TX (feeding N3 behavio*)) OR (TX (diet* N3 (restrict* or restrain* or reduc*))) OR (TX (waist* N3 circumferenc*)))
AND
((MH "Acceptance and Commitment Therapy") OR (TX (acceptance* N3 (commit* or mind* or base* or focus* or intervention* or therap* or treat*))))
Limiters - Published Date: 20190801-20220631

For peer review only

2.0 Data extraction form

2.1 Top-line details

ID of data extractor	
Covidence ID	
References (Author, Publication year, Titles)	
Trial ID (if provided)	

2.2 Study characteristics

2.2.1 Methods

Design		
Study start date		
Study end date		
Recruitment i.e. where and how recruited (online, proactive telephone etc)		
Setting (e.g. clinical, commercial, workplace, etc.)		
Country		
Inclusion/exclusion criteria	Inclusion criteria	
	Exclusion criteria	
Inclusion based on specific population characteristic? (e.g. pre-existing condition, gender, ethnicity)		

2.2.2 Notes

Funding statement (copy verbatim)	
Declarations of interest statement (copy verbatim)	
Any other notes to be included in characteristics of included studies table	

For peer review only

2.2.3 Participants (at baseline)

Total N randomized	
N per arm/group (where relevant)	
Total % Female	
Total mean age	
Total mean baseline BMI	
Total mean baseline EE (emotional eating)	
Total mean baseline UE (uncontrolled eating/ disinhibition)	
Total mean baseline R (restraint)	
Total mean baseline other EBTs (rename as appropriate)	

For peer review only

2.2.4 Interventions

(where multiple arms, copy and paste for each arm)

Any shared aspects between intervention and control groups	Target behaviour (e.g. physical activity or nutrition)	
	Mode of Delivery (e.g. group vs individual, online vs in person)	
	Duration & Frequency & Intensity (Dose)	
	Shared Intervention Content/ Components	
Comparison Arm (rename as needed)	Target behaviour (e.g. physical activity or nutrition)	
	Mode of Delivery (e.g. group vs individual, online vs in person)	
	Duration & Frequency & Intensity (Dose)	
	Behaviour change strategies	
	Other intervention details	
Intervention Arm (rename as needed)	Target behaviour (e.g. physical activity or nutrition)	
	Mode of Delivery (e.g. group vs individual, online vs in person)	

	Duration & Frequency & Intensity (Dose)	
	Behaviour change strategies	
	ACT components (add key terms, e.g. cognitive defusion, urge surfing, values, etc.)	
	Other intervention details	

2.3 Mediators/Moderators/Outcomes

How EBTs were measured/defined (which questionnaire was used, with citation)	
When EBTs were measured	
How weight outcome was measured (e.g. self-report vs objective etc.)	
When outcome was measured	

For peer review only

PRISMA-IPD Checklist of items to include when reporting a systematic review and meta-analysis of individual participant data (IPD)

PRISMA-IPD Section/topic	Item No	Checklist item	Reported on page
**Title**			
Title	1	Identify the report as a systematic review and meta-analysis of individual participant data.	1
**Abstract**			
Structured summary	2	Provide a structured summary including as applicable:	2
		**Background**: state research question and main objectives, with information on participants, interventions, comparators and outcomes.	
		**Methods**: report eligibility criteria; data sources including dates of last bibliographic search or elicitation, noting that IPD were sought; methods of assessing risk of bias.	
		**Results**: provide number and type of studies and participants identified and number (%) obtained; summary effect estimates for main outcomes (benefits and harms) with confidence intervals and measures of statistical heterogeneity. Describe the direction and size of summary effects in terms meaningful to those who would put findings into practice.	
		**Discussion:** state main strengths and limitations of the evidence, general interpretation of the results and any important implications.	
		**Other:** report primary funding source, registration number and registry name for the systematic review and IPD meta-analysis.	
**Introduction**			
Rationale	3	Describe the rationale for the review in the context of what is already known.	4
Objectives	4	Provide an explicit statement of the questions being addressed with reference, as applicable, to participants, interventions, comparisons, outcomes and study design (PICOS). Include any hypotheses that relate to particular types of participant-level subgroups.	5-6
**Methods**			
Protocol and registration	5	Indicate if a protocol exists and where it can be accessed. If available, provide registration information including registration number and registry name. Provide publication details, if applicable.	Na (PROSPERO registration on page 1)
Eligibility criteria	6	Specify inclusion and exclusion criteria including those relating to participants, interventions, comparisons, outcomes, study design and characteristics (e.g. years when conducted, required minimum follow-up). Note whether these were applied at the study or individual level i.e. whether eligible participants were included (and ineligible participants excluded) from a study that included a wider population than specified by the review inclusion criteria. The rationale for	6

For peer review only - http://bmjopen.bmj.com/site/about/guidelines.xhtml

		criteria should be stated.	
Identifying studies - information sources	7	Describe all methods of identifying published and unpublished studies including, as applicable: which bibliographic databases were searched with dates of coverage; details of any hand searching including of conference proceedings; use of study registers and agency or company databases; contact with the original research team and experts in the field; open adverts and surveys. Give the date of last search or elicitation.	6-7
Identifying studies - search	8	Present the full electronic search strategy for at least one database, including any limits used, such that it could be repeated.	Supplementary material
Study selection processes	9	State the process for determining which studies were eligible for inclusion.	7
Data collection processes	10	Describe how IPD were requested, collected and managed, including any processes for querying and confirming data with investigators. If IPD were not sought from any eligible study, the reason for this should be stated (for each such study).	7-9
		If applicable, describe how any studies for which IPD were not available were dealt with. This should include whether, how and what aggregate data were sought or extracted from study reports and publications (such as extracting data independently in duplicate) and any processes for obtaining and confirming these data with investigators.	
Data items	11	Describe how the information and variables to be collected were chosen. List and define all study level and participant level data that were sought, including baseline and follow-up information. If applicable, describe methods of standardising or translating variables within the IPD datasets to ensure common scales or measurements across studies.	7
IPD integrity	A1	Describe what aspects of IPD were subject to data checking (such as sequence generation, data consistency and completeness, baseline imbalance) and how this was done.	8-9
Risk of bias assessment in individual studies.	12	Describe methods used to assess risk of bias in the individual studies and whether this was applied separately for each outcome. If applicable, describe how findings of IPD checking were used to inform the assessment. Report if and how risk of bias assessment was used in any data synthesis.	7
Specification of outcomes and effect measures	13	State all treatment comparisons of interests. State all outcomes addressed and define them in detail. State whether they were pre-specified for the review and, if applicable, whether they were primary/main or secondary/additional outcomes. Give the principal measures of effect (such as risk ratio, hazard ratio, difference in means) used for each outcome.	9-11
Synthesis methods	14	Describe the meta-analysis methods used to synthesise IPD. Specify any statistical methods and models used. Issues should include (but are not restricted to):<br>• Use of a one-stage or two-stage approach.<br>• How effect estimates were generated separately within each study and combined across studies (where applicable).<br>• Specification of one-stage models (where applicable) including how clustering of patients within studies was accounted for.	9-11

For peer review only

		• Use of fixed or random effects models and any other model assumptions, such as proportional hazards.<br>• How (summary) survival curves were generated (where applicable).<br>• Methods for quantifying statistical heterogeneity (such as I^2 and τ^2).<br>• How studies providing IPD and not providing IPD were analysed together (where applicable).<br>• How missing data within the IPD were dealt with (where applicable).	
Exploration of variation in effects	A2	If applicable, describe any methods used to explore variation in effects by study or participant level characteristics (such as estimation of interactions between effect and covariates). State all participant-level characteristics that were analysed as potential effect modifiers, and whether these were pre-specified.	9-11
Risk of bias across studies	15	Specify any assessment of risk of bias relating to the accumulated body of evidence, including any pertaining to not obtaining IPD for particular studies, outcomes or other variables.	7
Additional analyses	16	Describe methods of any additional analyses, including sensitivity analyses. State which of these were pre-specified.	11
**Results**			
Study selection and IPD obtained	17	Give numbers of studies screened, assessed for eligibility, and included in the systematic review with reasons for exclusions at each stage. Indicate the number of studies and participants for which IPD were sought and for which IPD were obtained. For those studies where IPD were not available, give the numbers of studies and participants for which aggregate data were available. Report reasons for non-availability of IPD. Include a flow diagram.	na
Study characteristics	18	For each study, present information on key study and participant characteristics (such as description of interventions, numbers of participants, demographic data, unavailability of outcomes, funding source, and if applicable duration of follow-up). Provide (main) citations for each study. Where applicable, also report similar study characteristics for any studies not providing IPD.	na
IPD integrity	A3	Report any important issues identified in checking IPD or state that there were none.	na
Risk of bias within studies	19	Present data on risk of bias assessments. If applicable, describe whether data checking led to the up-weighting or down-weighting of these assessments. Consider how any potential bias impacts on the robustness of meta-analysis conclusions.	na
Results of individual studies	20	For each comparison and for each main outcome (benefit or harm), for each individual study report the number of eligible participants for which data were obtained and show simple summary data for each intervention group (including, where applicable, the number of events), effect estimates and confidence intervals. These may be tabulated or included on a forest plot.	na
Results of syntheses	21	Present summary effects for each meta-analysis undertaken, including confidence intervals and measures of statistical heterogeneity. State whether the analysis was pre-specified, and report the numbers of studies and participants and, where applicable, the number of events on which it is based.	na

For peer review only

		When exploring variation in effects due to patient or study characteristics, present summary interaction estimates for each characteristic examined, including confidence intervals and measures of statistical heterogeneity. State whether the analysis was pre-specified. State whether any interaction is consistent across trials.	
		Provide a description of the direction and size of effect in terms meaningful to those who would put findings into practice.	
Risk of bias across studies	22	Present results of any assessment of risk of bias relating to the accumulated body of evidence, including any pertaining to the availability and representativeness of available studies, outcomes or other variables.	na
Additional analyses	23	Give results of any additional analyses (e.g. sensitivity analyses). If applicable, this should also include any analyses that incorporate aggregate data for studies that do not have IPD. If applicable, summarise the main meta-analysis results following the inclusion or exclusion of studies for which IPD were not available.	na
**Discussion**			
Summary of evidence	24	Summarise the main findings, including the strength of evidence for each main outcome.	na
Strengths and limitations	25	Discuss any important strengths and limitations of the evidence including the benefits of access to IPD and any limitations arising from IPD that were not available.	na
Conclusions	26	Provide a general interpretation of the findings in the context of other evidence.	na
Implications	A4	Consider relevance to key groups (such as policy makers, service providers and service users). Consider implications for future research.	na
**Funding**			
Funding	27	Describe sources of funding and other support (such as supply of IPD), and the role in the systematic review of those providing such support.	15

A1 – A3 denote new items that are additional to standard PRISMA items. A4 has been created as a result of re-arranging content of the standard PRISMA statement to suit the way that systematic review IPD meta-analyses are reported.

© Reproduced with permission of the PRISMA IPD Group, which encourages sharing and reuse for non-commercial purposes

For peer review only

For peer review only
